# Local 3D matrix microenvironment regulates cell migration through spatiotemporal dynamics of contractility-dependent adhesions

**DOI:** 10.1038/ncomms9720

**Published:** 2015-11-09

**Authors:** Andrew D. Doyle, Nicole Carvajal, Albert Jin, Kazue Matsumoto, Kenneth M. Yamada

**Affiliations:** 1 Laboratory of Cell and Developmental Biology, Cell Biology Section, National Institute of Dental and Craniofacial Research, National Institutes of Health, Bethesda, Maryland 20892, USA; 2 Laboratory of Cellular Imaging and Macromolecular Biophysics, National Institute of Biomedical Imaging and Bioengineering, National Institutes of Health, Bethesda, Maryland 20892, USA

## Abstract

The physical properties of two-dimensional (2D) extracellular matrices (ECMs) modulate cell adhesion dynamics and motility, but little is known about the roles of local microenvironmental differences in three-dimensional (3D) ECMs. Here we generate 3D collagen gels of varying matrix microarchitectures to characterize their regulation of 3D adhesion dynamics and cell migration. ECMs containing bundled fibrils demonstrate enhanced local adhesion-scale stiffness and increased adhesion stability through balanced ECM/adhesion coupling, whereas highly pliable reticular matrices promote adhesion retraction. 3D adhesion dynamics are locally regulated by ECM rigidity together with integrin/ECM association and myosin II contractility. Unlike 2D migration, abrogating contractility stalls 3D migration regardless of ECM pore size. We find force is not required for clustering of activated integrins on 3D native collagen fibrils. We propose that efficient 3D migration requires local balancing of contractility with ECM stiffness to stabilize adhesions, which facilitates the detachment of activated integrins from ECM fibrils.

## Abstract (web summary)

Little is known about how the physical properties of three dimensional (3D) extracellular matrices modulate cell adhesion dynamics. Here Doyle *et al.* generate 3D collagen gels of varying microarchitecture and quantify the effect on adhesion dynamics and cell motility.

## Graphical abstract

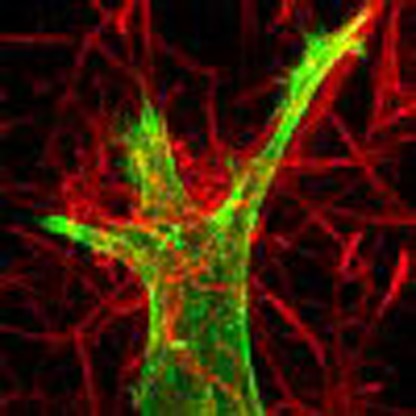

Cell interactions with the surrounding microenvironment regulate key intracellular processes, including signalling cascades, gene regulation, and cell fate^1^. This type of ‘outside-in’ signalling includes physical signals from the extracellular matrix (ECM) that can profoundly affect cell migration. The physical characteristics of an ECM, including stiffness, composition, and topography, can regulate migration^2^,^3^,^4^. While cell migration is considered a cyclic process consisting of (1) protrusion, (2) adhesion, (3) translocation and (4) retraction^5^,^6^, it is the direct coupling between the cell and the ECM at integrin-based adhesion sites that permits cells to mechanically sense their physical surroundings and adjust mechanisms of migration^7^,^8^,^9^,^10^.

On flat 2D surfaces, adhesion formation and maturation are well characterized and provide a physical link between the actin cytoskeleton and the ECM. Following integrin ligation, cell adhesion components accumulate to form focal complex/nascent adhesions, which undergo growth and maturation into focal adhesions after linking to F-actin structures^11^,^12^. Adhesions are proposed to work as a molecular clutch, where forces are transferred between the ECM and the cytoskeleton, as demonstrated by the differential turnover of various adhesion proteins^7^,^8^,^13^. Previous research has established that local force application can promote cell adhesion growth, maturation, and stability while bound to the ECM^13^,^14^. This depends on a number of adhesion proteins that respond to mechanical stress^10^,^15^, acting together with integrins and myosin II, which show enhanced affinity towards their ligands under tension^16^,^17^. Elevated contractile force can lead to adhesion disassembly through retraction^14^, and loss of myosin II contractility results in immediate adhesion disassembly^18^. These features suggest that forces must be balanced for stabilization of adhesions^19^. Adhesion stability also depends on the microenvironment: adhesions in 3D cell-derived matrices and on one-dimensional fibrillar patterns are more stable than on 2D substrates^20^. Thus, adhesion sites represent a dynamic, ever-changing physical link between cellular contractile elements and the external environment that require a proper balance of contractile forces to match local environmental conditions.

Whereas cell migration on 2D substrates has been extensively characterized, 3D migration is poorly understood because of the physical complexity of the local microenvironment. Beyond the ECM physical regulators known for 2D migration (stiffness, ligand density and composition), a 3D matrix adds ECM microarchitecture, porosity, and elastic behaviour^2^. Wolf *et al.*^4^ established that increased collagen gel porosity permits more rapid migration of cancer and immune cells, with decreased need for ECM proteolysis. Chemical stiffening of a 3D ECM impedes cell invasion, matrix remodelling and cell migration^21^, whereas aligned topographies enhance cell migration^22^,^23^. Although the physical 3D environment can regulate cell migration, little is known about the dynamics of 3D ECM-cell adhesion sites. Kubow *et al.*^24^ found that 3D cell adhesion length is determined by local alignment along collagen or electrospun fibres and is dependent on myosin II contractility. However, it is not clear how local differences in 3D matrix stiffness and other ECM physical factors affect the mechanical behaviour of 3D adhesions and cell migration.

In this study, we examine how the physical attributes of 3D matrix regulate the mechanics of cell adhesions and their role in 3D cell migration. By changing the local physical characteristics of type I collagen gels to mimic a more *in vivo*-like environment, we find that local fibre stiffness can vary by up to 10-fold. These microenvironmental ECM differences alter 3D adhesion protein turnover and overall adhesion maturation and stability through myosin II contractility and integrin activation. Abrogation of contractility reduces fibroblast 3D migration regardless of ECM porosity and uncouples leading edge and cell body movements; unexpectedly high integrin activation in 3D matrix impedes cell body translocation. Our results suggest that regulation of 3D cell adhesion dynamics through the ECM plays a major role in 3D cell migration.

## Results

### Adhesion-level differences in ECM structure and stiffness

**Figure 1:**
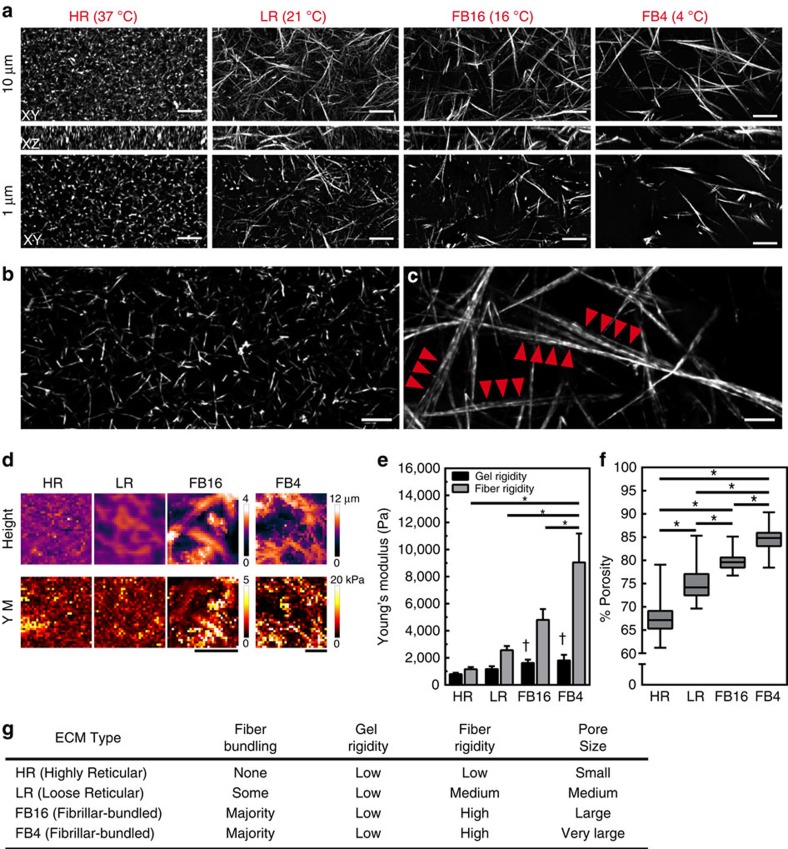
3D collagen gel heterogeneity is associated with local ECM stiffening. (**a**) Ten-micrometre maximum intensity projection (MIP) XY (top), 10 μm XZ (middle), and 1 μm MIP XY (bottom) images of 3 mg ml^−1^ rat-tail collagen polymerized at different temperatures (37, 21, 16 and 4 °C) illustrating diverse ECM architectures: highly reticular (HR), loose reticular (LR), and fibrillar bundlled (FB). (**b**,**c**) Structured illumination microscopy of HR (**b**) and FB16 (**c**) collagen (2 μm MIP). Arrowheads in **c** indicate aligned bundlled fibrils not found in HR or LR ECMs. (**d**) Representative examples of AFM-generated local height (top) and Young’s modulus (YM) force-volume (bottom) maps for all ECM conditions. (**e**) Average Young’s modulus for gels and fibrils in each ECM condition; *N*=3, *n*=9. (**f**) Percent porosity of a 4-μm thick section of collagen for all ECM conditions. *N*=3, *n*>25. (**g**) Reference table summarizing the major structural features of each ECM condition. *P<0.05, significantly different. † Significantly different fibre rigidity in the same ECM condition, *P*<0.05 (ANOVA). Errors bars: s.e.m. Scale bars: (**a**) 10 μm; (**b**,**c**) 2 μm; (**d**) 20 μm.

To determine the impact of ECM structure on cell migration, we polymerized rat tail collagen gels at different temperatures (37, 21, 16 and 4 °C) to generate 3D ECM architectures with varying physical characteristics, which was previously shown to alter the physical organization of the entire gel^4^,^25^ (Fig. 1). At lower polymerization temperatures, gel porosity increased and the gel lattice became more heterogeneous than highly reticular (HR) 37 °C-polymerized collagen, while fibre diameter appeared to increase (Fig. 1a,f, and Supplementary Fig. 1a). Structured illumination microscopy discovered this change in diameter to be the bundling of multiple fibrils (Fig. 1b,c, Supplementary Movies 1–3). This fibrillar bundlled (FB) architecture was not observed in the HR condition and seldom in loose-reticular (LR) 21 °C collagen (Fig. 1a–c). Analysis of fibril diameter revealed that HR matrices were composed of only single fibrils averaging ∼310 nm in diameter. Conversely, FB16 matrices contained ∼10% single fibrils of similar size along with bundlled fibrils that together averaged 899 nm. Bundles could consist of 2 to as many as 12 or more individual fibrils. Consequently, fibril bundling and not fibre thickening was responsible for the structural differences we observed. Although fibril lengths were difficult to assess due to the extensive fibril overlap in HR ECMs, FB16 ECMs demonstrated a near sevenfold difference in length (HR: 3.6±1.4 s.d. μm; FB16: 21.9±7.6 μm, Supplementary Fig. 1b).

We next examined whether these fibril architectures (highly reticular and fibrillar bundled) were present *in vivo*. Immunostaining for type I collagen demonstrated that all of the types and bundle diameters of fibrils we generated *in vitro* were present in adult mouse ear and embryonic day 17 mouse back skin (Supplementary Fig. 1c,d), often located adjacent to each other as previously reported^26^. These data indicate that *in vitro* HR and FB collagen gels with controlled differences in architecture can be used as an alternative to the very heterogeneous *in vivo* fibril architectures to analyse cellular mechanical interactions with specific features of the 3D microenvironment.

These structural differences led us to investigate effects on gel stiffness. We sought to observe and measure ECM stiffness at the size scale at which a cell mechanically interacts with the ECM, performing atomic force microscopy (AFM) using conical-tipped pyramidal cantilevers at 1 μm resolution that are similar in size to a single-focal adhesion^27^. Raster-scanning of collagen gel surfaces provided detailed height and Young’s modulus measurements from force-volume maps (Fig. 1d). At the scale of a cell (32 × 32 μm), the ECMs showed no significant differences in gel stiffness (Fig. 1e) with values similar to previous reports using gels >2 mg ml^−1^ (ref. 4). However, at the scale of cell adhesions, the increased bundle thickness and overall fibre heterogeneity of FB collagen was associated with large variations in local fibre rigidity (Fig. 1d–g). This discrepancy could be attributed to differing porosities between ECM types. Exclusion of pore areas from AFM measurements revealed significant elevation of fibril-associated stiffness for ECMs containing bundled fibrils (FB16: threefold; FB4: fivefold; Fig. 1e). To confirm differences in stiffness at the cell adhesion versus cellular size scales, we performed AFM measurement using a 38-μm diameter bead. Such cell-scale measurements showed reduced stiffness for heterogeneous FB16 and FB4 matrices (Supplementary Fig. 1e). Thus, macro/cell scale rigidity measurements can miss additional differences in local fibre stiffness relevant to single adhesions, likely due to differences in pore size and local gel heterogeneity. For reference, these ECM differences are summarized in Fig. 1g.

### ECM architectures influence fibroblast 3D migration

**Figure 2:**
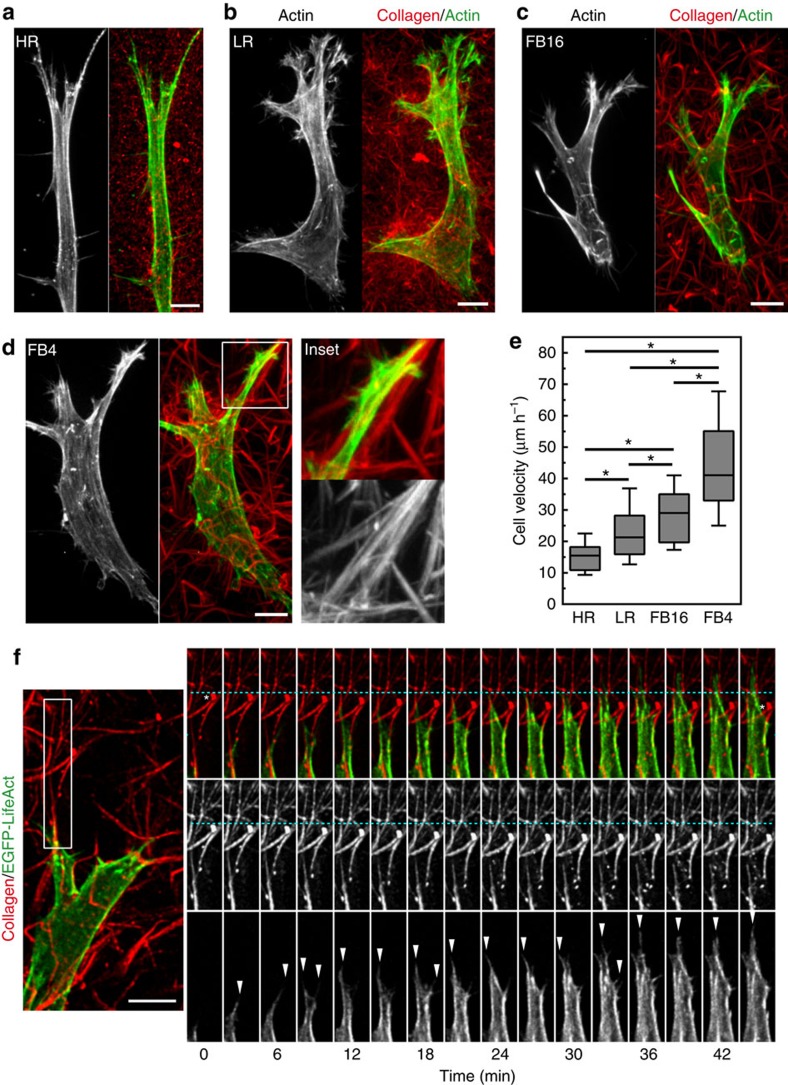
Migratory differences and similarities as fibroblast traverse different ECM architectures. (**a**–**d**) MIP images of HFFs for actin (green) within Atto-647N labelled collagen gels (red) demonstrates cell morphology differences associated with HR, LR, FB16 and FB4 ECMs. Inset (white box in **d**) illustrates pseudopodial extension along bundled collagen fibrils in FB4 ECM. Note the changes in cell morphology with changes in the ECMs. (**e**) Fibroblast migration velocity for each ECM. *N*>3 replicates, *n*>60. Errors bars: s.e.m. (**f**) Live-cell microscopy of a fibroblast expressing EGFP-Lifeact (green or grayscale) within FB4 ECM (red). Timelapse montage (right panels) illustrates the formation of actin-rich filopodial structures (arrowheads) forming along bundled collagen fibrils, directing migration. White asterisks indicate the initial and final positions of collagen fibrils relative to a fiduciary line (cyan) after cellular contraction pulls the matrix towards the cell body, while the leading edge protrudes forward. *P<0.05 (ANOVA), *N*≥3, *n*≥70 for all conditions. Scale bars, 10 μm.

The diverse physical properties of these ECMs led us to examine their impact on fibroblast morphology and migration. Human foreskin fibroblasts (HFFs) showed spindle-shaped morphologies typical of 3D ECMs (Fig. 2a–d), with fewer lateral processes in HR ECMs. Timelapse microscopy and cell tracking confirmed that cell migration rates rose with increasing ECM porosity (Fig. 2e)^4^. Live-cell spinning disk microscopy of EGFP-Lifeact revealed altered protrusions in the presence of bundled fibrils: leading-edge protrusions often followed the fibre along its length. This contact-guidance effect was particularly noticeable when fibrils were parallel to the direction of the protrusions (Fig. 2d,f). However, at the cellular scale, fibroblasts displayed similar migratory phenotypes in the different ECMs, pulling anterior collagen fibrils retrograde towards the cell body (Fig. 2f, Supplementary Movie 4). Matrix near the cell body was pulled anterograde, suggesting a contractile axis between the leading and trailing edges. Altogether, these data indicate that different matrix architectures can influence cell migration rates, though fibroblasts employ a similar migratory process for pulling the cell body through 3D space.

### ECM stiffness alters adhesion turnover but not components

**Figure 3:**
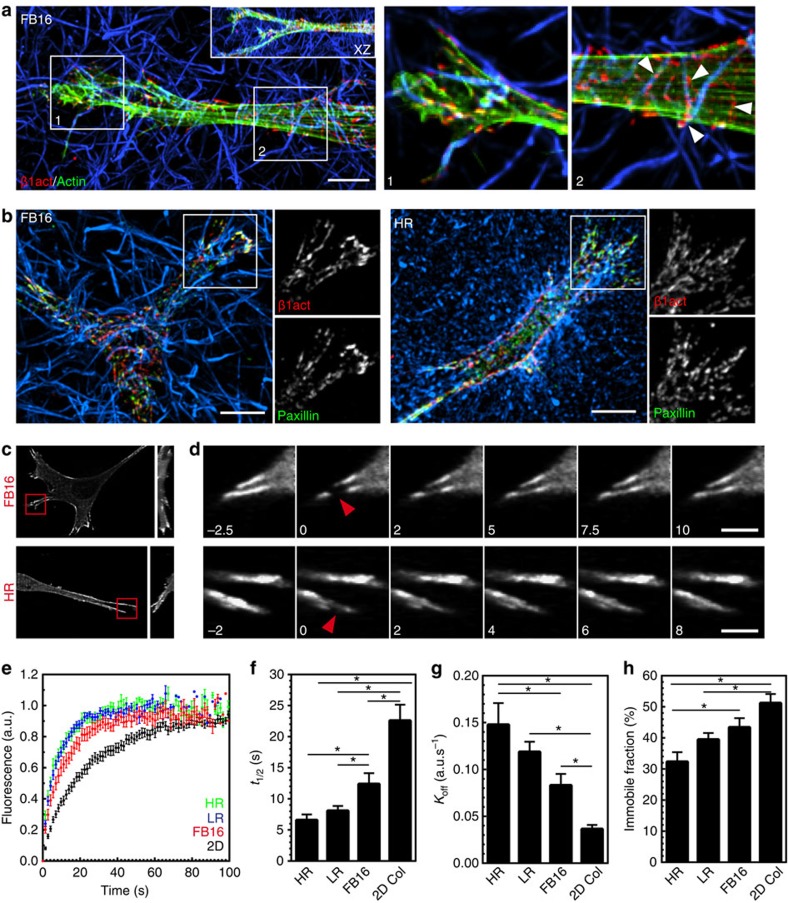
ECM fibre stiffness alters adhesion turnover but not adhesion components. (**a**) Full MIP localizing activated β1 integrin (9EG7: red) and phalloidin staining for F-actin (green) in FB16 ECMs. Inset shows XZ projection. Panels 1 and 2 illustrate integrins following the contours of the ECM and not stress fibre arrangement (indicated by arrowheads). Panels 1 and 2 are partial Z projections of the cell region. (**b**) Localization of activated β1 integrin (9EG7: red) and paxillin (green) in FB16 (left) and HR collagen matrices (right). Insets show variable colocalization that is prominent at the leading edge. All images are maximum intensity Z projections of ∼30 μm. (**c**) MIP images showing eGFP–zyxin localization in HR or FB16 ECM acquired before FRAP. 3D YZ MIP shown on the right. (**d**) Timelapse sequence shown in **c** illustrating EGFP-zyxin FRAP kinetics. Red arrowheads indicate the FRAP site. Time is in seconds. (**e**–**h**) FRAP kinetic analysis showing *t*_1/2_ (**f**) *K*_off_ rates (**g**) and immobile fractions related to the graphs in E. *N*≥3, and *n*≥20 for all conditions. Errors bars: s.e.m. *P<0.05 (ANOVA). Scale bars: (**a**,**b**) 10 μm, (**d**) 5 μm.

Because adhesion sites represent the mechanical interface between a cell and the ECM, we investigated whether the composition and kinetics of 3D adhesions depend on the adhesion-scale differences in stiffness between the ECM architectures. Adhesion composition was not altered: immunostaining for activated β1 integrin (β1act) revealed integrin ligation at every cell/ECM contact along the cell length within all ECMs. In FB matrices, the actin stress fibres present in all ECM microarchitectures were aligned parallel to the cell’s long axis, but interestingly, β1act tended to align more with the ECM architecture than with the intracellular stress fibres (Fig. 3a). However, because of the highly reticular, closely packed fibril architecture in HR, such spatial orientation of β1act was difficult to discern in HR ECMs (Supplementary Fig. 2). Immunostaining for, or transfection with, fluorescent protein-tagged adhesion proteins demonstrated that talin, vinculin, tensin 1, zyxin and paxillin were present in similar distributions in the adhesive structures of all ECMs (Fig. 3b, Supplementary Fig. 3).

However, because adhesion sites are dynamic structures that respond to force application, differences in fibre stiffness could affect the kinetics/turnover of focal adhesion proteins involved in the molecular clutch^7^,^19^,^28^,^29^. To test this, we examined protein turnover via fluorescence recovery after photobleaching (FRAP) on EGFP–zyxin containing adhesions in the different 3D ECMs and on 2D collagen covalently linked to the coverslip (Fig. 3c–h). The data indicate higher local ECM stiffness (2D>FB16>LR>HR) stabilizes zyxin in adhesions according to binding (increased *τ*_1/2_) and dissociation (decreased *K*_off_) rates, along with increased immobile fraction (Fig. 3h). These adhesion kinetics data suggest that rigid fibrils promote the efficiency of the molecular clutch within adhesions, while compliant ECMs result in increased adhesion slipping with inefficient coupling.

### 3D adhesion maturation depends on ECM stiffness

**Figure 4:**
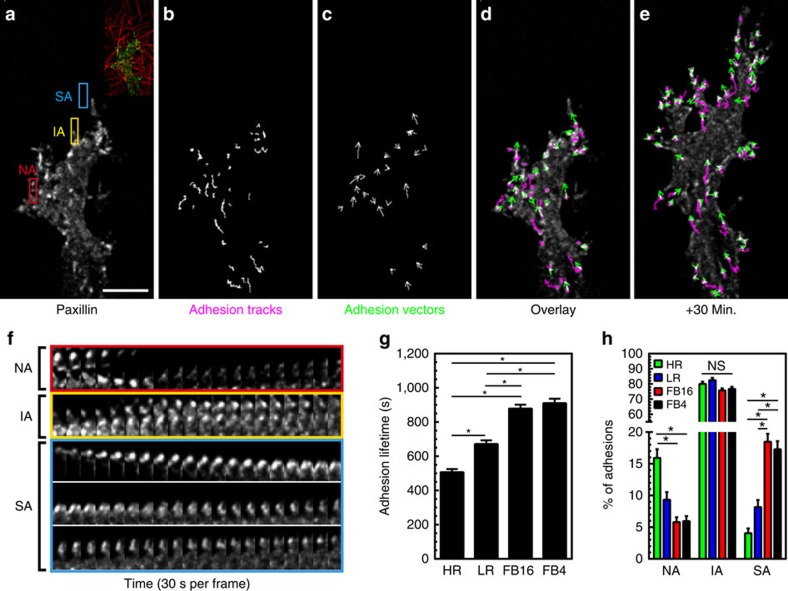
3D adhesion maturation depends on ECM stiffness. (**a**) MIP of EYFP-paxillin adhesions associating with FB4 collagen gels. Inset shows ECM structure. (**b**,**c**) Adhesion-tracking mapping of adhesion displacements over their lifetime, while adhesion vector mapping (**c**) illustrates their averaged instantaneous movement. (**d**,**e**) Overlay of EYFP-paxillin (white) adhesion tracks (magenta) and adhesion vectors (green) at the time point shown in **a** (**d**) and 30 min later (**e**). (**f**) Timelapse kymographs from the colour-coded boxes in **a** illustrating adhesions with different lifetimes. Red box: nascent adhesions (NA: 0–120 s.). Yellow box: intermediate adhesions (IA: 500–1,500 s.). Cyan box: stable adhesions (SA: >1,500 s.). Adhesion for cyan appears several frames after the initial timepoint. (**g**) Average lifetime of adhesions for each condition. (**h**) Percent of adhesions considered NA, IA, or SA in each ECM condition. *n*-values for (**g**,**h**) are >500 adhesions and a minimum of 6 cells per condition. Errors bars: s.e.m. *P<0.05 (ANOVA). Scale bar, 10 μm.

Local fibre stiffness might regulate not only the kinetics of adhesion components, but also the maturation and stability of entire adhesions. To quantify these events, we imaged HFFs expressing EYFP-paxillin and used automated adhesion tracking to determine adhesion lifetime (Fig. 4a–e)^30^,^31^. This characterized adhesion lifetimes for all adhesions within a cell (>50 per cell), as opposed to focusing on a relatively small subset, while providing a spatiotemporal vectoral map for each adhesion (Fig. 4c–e, Supplementary Movie 5). In every ECM architecture, individual adhesion lifetimes varied greatly from unstable nascent adhesions (NA: 60–120 s.) to mature and highly stable adhesions (SA: >1,500 s.; Fig. 4f–h). However, the numbers of nascent and mature adhesions were inversely related for HR and FB ECMs, leading to a stiffness-dependent increase in adhesion lifetime (Fig. 4g,h). Interestingly, the predominant adhesion subtype (termed intermediate adhesions: IA, 150–1,470 s.) showed <10% variation between HR and FB conditions. We next examined whether fibril diameter (>1 micron) or alignment of an adhesion along a fibril correlates best with adhesion stabilization in FB4 gels. Adhesions were preferentially stabilized parallel to collagen fibres versus on fibrils with diameters >1 μm (Supplementary Fig. 4a). Altogether, these data indicate that local fibril stiffness can determine adhesion lifetime by altering the relative proportion of nascent versus mature adhesions.

### Two 3D adhesion populations dependent on ECM/rigidity

**Figure 5:**
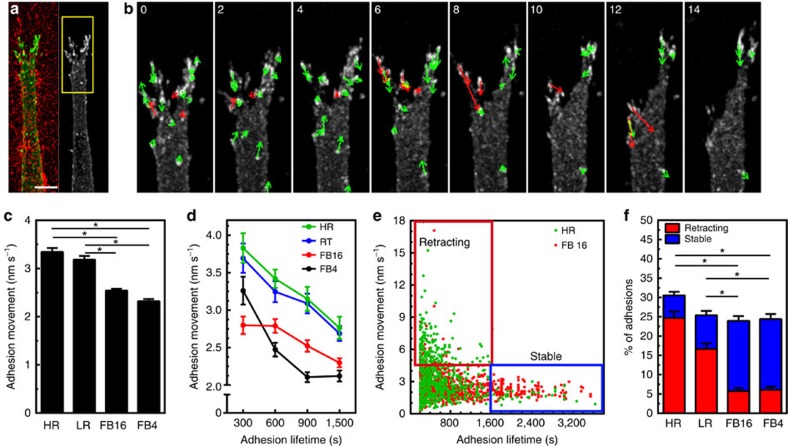
Identification of two distinct 3D adhesion populations dependent on ECM/rigidity. (**a**,**b**) Rapid movements of EYFP-paxillin adhesions in HR collagen. (**b**) Timelapse montage of region depicted in **a**. Arrows indicate direction of adhesion movement: Red arrows highlight adhesions undergoing rapid movement that retract away from the leading edge and slow migration. Time is in min. (**c**) Average movement of adhesions within the different ECMs. (**d**,**e**) Adhesion movement plotted against grouped adhesion lifetime (**d**) or as a scatterplot (**e**). Boxes in **e** show retracting (red) and stable (blue) adhesion populations. (**f**) Percent of adhesions that are retracting (red) or stable (blue) in each ECM. *n*-values for (**c**) through (**f**) are >500 adhesions and a minimum of six cells per condition. Errors bars: s.e.m. *P<0.05 (ANOVA). Scale bar, 10 μm.

Further examination of adhesion dynamics via adhesion vector mapping revealed that adhesion displacement was substantially lower within ECMs containing stiff bundled fibrils (FB gels; Fig. 5c). In fact, we found a strong inverse correlation between adhesion movement and their longevity in all matrices; short-lived adhesions demonstrated rapid movements, compared with slow movements of mature, stable adhesions (Fig. 5a–c,e, Supplementary Movie 6), thus identifying two distinct adhesion populations that varied according to the ECM (Fig. 5e,f, Supplementary Fig. 4b–e). By tracking each adhesion type with respect to the surrounding ECM, we found that highly mobile adhesions were in fact undergoing retraction, pulling away from collagen fibrils to which they were adhering and impeding further progression of the leading edge. Before detachment from the ECM, retracting adhesions exhibited rapid, repetitive movement away from matrix fibrils, which preceded uncoupling from the ECM (Fig. 5a,b; Supplementary Fig. 5; Supplementary Movies 7 and 8). In contrast, stable adhesions remained continually associated with, and moved along the adjacent ECM. This repetitive ‘matrix tugging’ was not observed from stable adhesions and may reflect the relative consistent local association between the cell and the matrix. Overall, the relative proportions of these two adhesion populations depended on the local microenvironment, where soft HR versus stiffer FB matrices showed high numbers of retracting versus stable adhesions, respectively (Fig. 5f). These observations indicate that ECM rigidity can locally stabilize 3D adhesions, which is reflected in the relative proportions of retracting and stable adhesions.

### Balanced adhesion is required for efficient 3D migration

**Figure 6:**
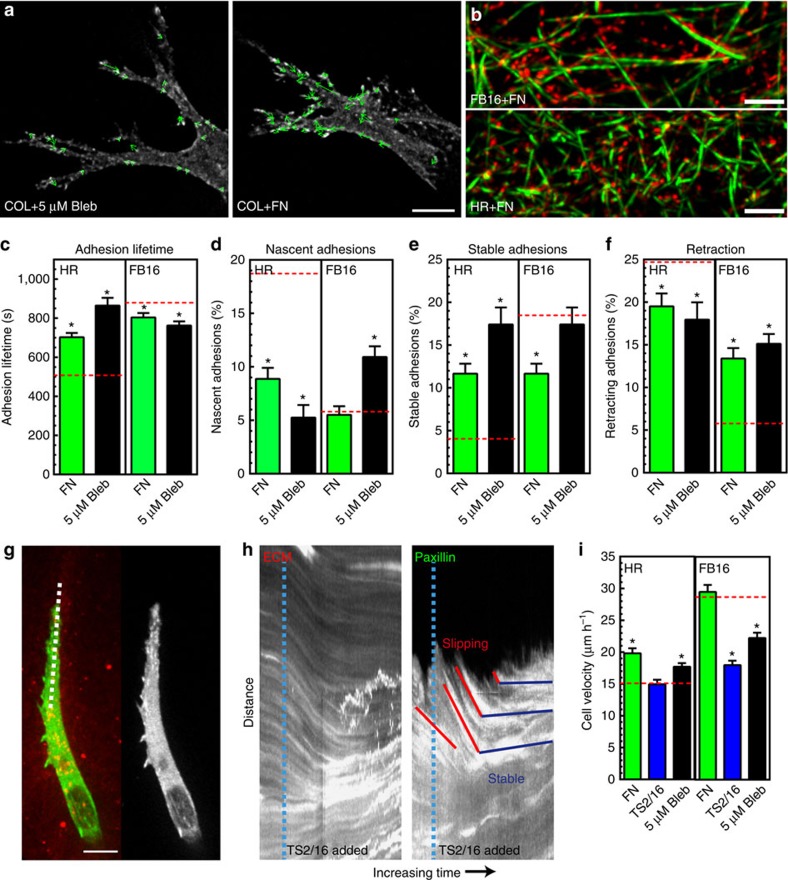
Balancing cytoskeletal tension with ECM/adhesion coupling is required for efficient 3D migration. (**a**) Adhesion movements in HR collagen+5 μM blebbistatin (Bleb; left) and HR collagen+FN (right). (**b**) SIM images of 300 μg ml^−1^ fibronectin (red) associated with FB16 or HR collagen fibrils (green). (**c**–**f**) Treatment of collagen with HPFN or reducing contractility with 5 μM blebbistatin has differential ECM-dependent effects on adhesion lifetime (**c**) nascent (**d**) and stable adhesions (**e**) and the population of retracting adhesions (**f**). Dashed red lines in (**c**–**f**) indicate untreated control levels (DMSO, Ig_G_). *N*≥3, *n*≥400. (**g**,**h**) Integrin activation (TS2/16; 2 μg ml^−1^) stabilizes adhesions. Kymographs ((**g**) dashed white line) show adhesion slippage and retraction (red lines) until the addition of TS2/16 antibody (dashed cyan lines in **h**). Antibody treatment activates integrins for ECM gripping and results in adhesion stabilization (horizontal blue lines). (**i**) Migration of HPFN, TS2/16, or 5 μM blebbistatin treated cells compared with controls *N*=3, *n*>48 (dashed red lines).* Significantly different from control ECM; *P*<0.05 (ANOVA). Errors bars: s.e.m. Scale bars: (**a**) 2 μm; (**b**,**g**) 10 μm.

Our data suggested that ECM/adhesion coupling is reduced in pliable HR ECMs and results in unstable adhesions and retraction. We hypothesized that this instability results from a contractility imbalance in which cellular contraction exceeds the threshold level of force that integrin/ligand binding can withstand at an individual adhesion site, so that the adhesion becomes unstable and retracts. To attempt to re-balance the level of contractility to maintain the local ECM coupling in HR matrices, we partially reduced contractility in cells within HR ECM using 5 μM blebbistatin. Traction force analysis confirmed that this lower dose of blebbistatin results on a moderate (∼50%) reduction in fibroblast contractility compared with a near-complete loss at 25 μM (Supplementary Fig. 6a–c). Analysis of 3D adhesion dynamics revealed differential ECM-dependent effects: adhesions formed in soft HR gels showed increased adhesion lifetimes, maturation and stabilization, with a reduced population of nascent and retracting adhesions (Supplementary Fig. 6d). Reducing contractility had the opposite effect on adhesion dynamics in stiff FB16 ECMs (Fig. 6c–f) and even led to retraction of highly stable adhesions (Supplementary Fig. 6e). Importantly, even though reduced contractility decreased overall cell migration for FB ECMs, it was significantly accelerated in HR conditions (Fig. 6i). These data support our hypothesis that an appropriate balance of contractility to local ECM compliance is needed at cell adhesions for their maturation/stabilization and efficient fibroblast migration. An informal analogy would be that highly pliable fibrils require a soft touch to allow adhesion stabilization, whereas stiffer bundled fibrils require higher cellular contractile forces to maintain adhesions.

### Integrin/ECM interactions impact 3D adhesion dynamics

While contractile force can greatly impact adhesion stabilization, regulation might also occur at the point of integrin/ECM ligation. The relative avidity of integrins for ligands could play a role because their interactions involve force-dependent catch-bonds^17^,^32^. To test this hypothesis, we altered integrin/ligand interactions in three ways: (1) treating preformed collagen gels containing fibroblasts with 300 μg ml^−1^ fibronectin (FN), its concentration in blood; (2) increasing integrin avidity via a stimulatory antibody (TS2/16); and (3) increasing integrin activation by the addition of manganese. Exogenous fibronectin became bound to collagen fibrils, covering most of the fibre surface (Fig. 6b). AFM confirmed that FN binding had no effect on collagen gel elasticity (HR=770±30 s.d. Pa: HR+FN=795±63 Pa, 3 replicates, *n*=12). Addition of FN substantially increased adhesion lifetime in HR gels by reducing nascent/retracting adhesions, and it enhanced cell migration (Fig. 6d,f,i). This mimicked the effect of reducing contractile force, even though adhesion movement was increased (Supplementary Fig. 6f). In contrast, FN treatment of FB16 ECMs produced small but significant reductions in adhesion lifetime with increased numbers of retracting adhesions (without altering nascent adhesions), and cell migration was enhanced. Stimulating ECM/adhesion coupling by treating cells with TS2/16 (2 μg  ml^−1^) showed similar effects as FN on cells in both ECMs. Kymograph analyses illustrate that shortly after TS2/16 addition cells dramatically grip the matrix (Fig. 6g,h). However, instead of inducing leading edge retraction, adhesions were stabilized in both ECM conditions. This enhanced stability caused no change in HR 3D migration, but significantly reduced migration through FB ECMs. A similar effect was found for fibroblasts treated with 50 μM MnCl_2_ (Supplementary Fig. 7a). Altogether, these data point to key roles for dynamic interactions between integrins and ligands in regulating 3D adhesion stability, which must be balanced with contractility for efficient migration.

### Contractility regulates 3D migration regardless of pore size

**Figure 7:**
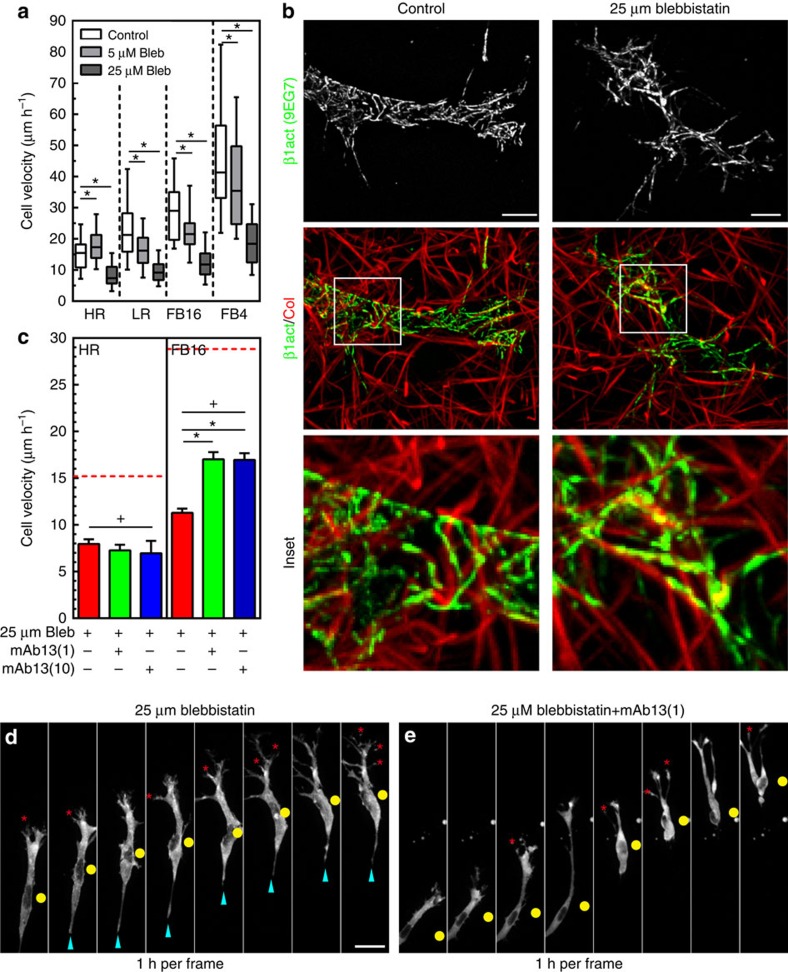
Myosin II contractility regulates 3D migration independent of pore size. (**a**) Fibroblast 3D migration rates for control (white) 5 μM (light grey) and 25 μM (dark grey) blebbistatin for each ECM architecture. *N*=3, *n*=48. (**b**) MIPs comparing activated β1 integrin (9EG7) in fibroblasts between control and overnight exposure to 25 μM blebbistatin in FB4 collagen. Inset illustrates that activated β1 integrin remains associated with collagen fibrils surrounding the cell body region in the absence of myosin II contractility. (**c**) Reducing integrin binding with an inhibitory antibody against β1 integrin (mAb13; 1 and 10 μg ml^−1^) partially rescues contractility-deficient migration, but in a pore size-dependent manner. Red dashed lines indicate control levels. *N*=3, *n*≥70.(**d**) Timelapse series of a fibroblast expressing EYFP-paxillin in FB16 ECM treated with 25 μM blebbistatin (added 30 min before first frame). Numerous protrusions (red asterisks) form but do not aid cell body movement (yellow circle), while an elongated tail (cyan arrowheads) hinders migration. (**e**) HFF treated with blebbistatin and mAb13 (1 μg ml^−1^) show fewer protrusions while showing no elongated tails and slipping through the matrix. *P<0.05 (ANOVA). + Significantly different from control conditions; *P*<0.05 (ANOVA). Errors bars: s.e.m. Scale bars: (**b**) 10 μm; (**d**,**e**) 30 μm.

Our results showing differing force requirements for adhesion stabilization and migration within different ECMs led us to ask whether a complete loss of contractility would result in similar differential ECM-dependent effects. However, treatment of fibroblasts with 25 μM blebbistatin reduced 3D migration rates in all ECM conditions (Fig. 7a) and led to simultaneous increases in protrusions, initiated ECM relaxation, and slowed cell body movement (Fig. 7d and Supplementary Movie 9). Interestingly, immunostaining revealed activated β1 integrins remained clustered at low force (5 μM blebbistatin; Supplementary Fig. 7b) or even in the complete absence of force (25 μM blebbistatin; Fig. 7b, Supplementary Fig. 7c) in all ECM conditions. Such constitutively engaged integrins might impede 3D migration unless disengaged by active contraction applied to adhesion sites. We tested this hypothesis by treating HFFs within HR and FB16 gels with mAb13 to reduce β1 integrin binding (1 and 10 μg ml^−1^) together with 25 μM blebbistatin. Reducing integrin binding partially rescued 3D cell migration in large-pore gels (FB), allowing cells to slip through the matrix (Fig. 7c,e, and Supplementary Movie 10), while HR still demonstrated reduced migration consistent with pore size dependence. Altogether, these data suggested a requirement for active contraction to physically detach clustered integrins during fibroblast 3D migration.

**Figure 8:**
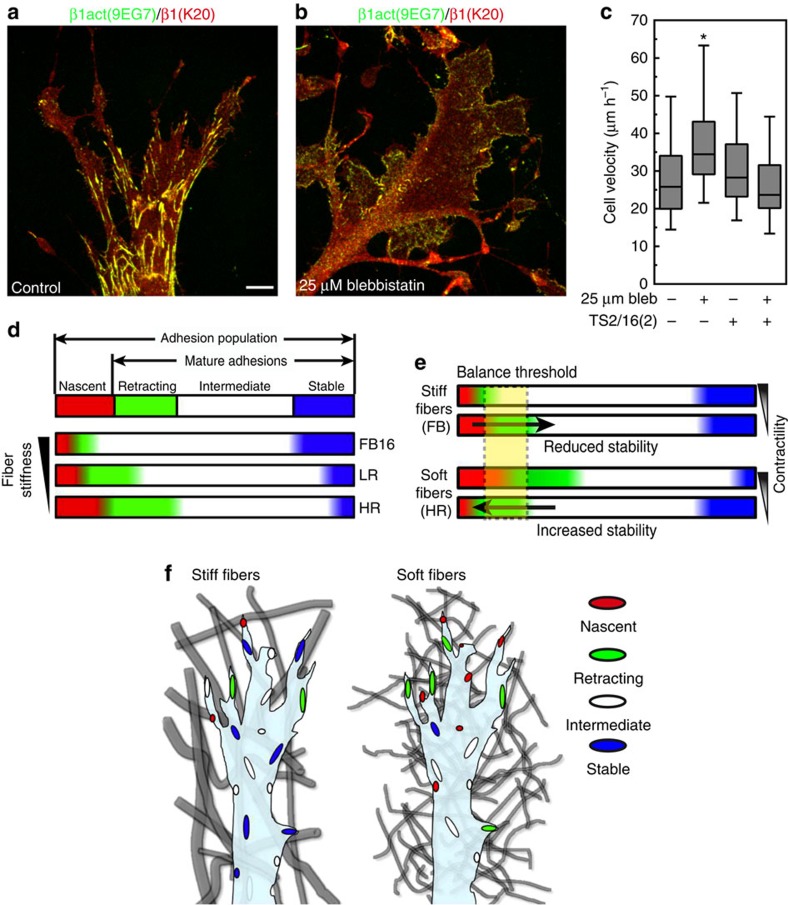
Contractility independent increase in 2D migration is due to a concomitant decrease in integrin activation. (**a**,**b**) Comparison of control (**a**) and treatment with 25 μM blebbistatin (**b**) on 2D collagen (50 μg ml^−1^) shows a lack of activated β1 integrin clustering (green) compared with K20 anti-total β1 integrin antibody (red). (**c**) Addition of the activating β1 integrin antibody TS2/16 (2 μg ml^−1^) to fibroblasts migrating across 2D globular collagen inhibits the increase in migration associated with 25 μM blebbistatin, *N*=3, *n*>80 (ANOVA). Errors bars: s.e.m. (**d**) Schematic summary of regulation by the 3D microenvironment of the dynamics of cell adhesions. The adhesion population consists of nascent (red) and mature adhesions, the latter comprised of retracting (green) intermediate (white), or stable (blue). As ECM fibre stiffness increases (bottom to top) the relative number of nascent and retracting adhesions are reduced, while highly stable adhesions are promoted. HR, LR and FB16 adhesion populations illustrate the actual calculated percentages for each condition. (**e**) Reducing the relative contractility of cells shifts the balance of nascent and retracting adhesions differently in soft (HR) and stiff (FB) ECMs, promoting further adhesion stability in the former. The critical balance threshold is shown in yellow. Arrows indicate change in the adhesion population above or below the threshold. (**f**) Schematic representation of the adhesion populations for cells within heterogeneous matrix of stiff fibrils (left) and homogeneous matrix of soft fibrils (right). Adhesion colour depicts the adhesion type shown in **d**. *Significantly different from all other conditions; *P*<0.05 (ANOVA). Scale bar, 10 μm.

Consequently, the requirement for contractility in 3D migration may involve this elevated integrin clustering. On 2D globular collagen, blebbistatin treatment increases migration but reduces integrin activation and clustering (Fig. 8a–c). To resolve this discrepancy, we artificially activated integrins on 2D globular collagen. Alone, activating β1 antibody TS2/16 did not affect 2D migration, but simultaneous integrin activation and blebbistatin inhibition reduced 2D migration rates back to control levels (Fig. 8c). Therefore, the differences in contractility-dependence of 2D versus 3D fibroblast migration can be explained by enhanced integrin activation and clustering on native 3D ECM structures.

## Discussion

Here we describe how the local microenvironment determines 3D adhesion maturation and regulates cell motility. The local structural differences in heterogeneous matrices containing bundled fibrils result in large variations in fibre stiffness, which we find alters the mechanical efficiency and coupling between individual adhesions and local ECM structures to modulate adhesion stabilization. In addition, we identified a novel concept clarifying the requirement for contractile force in fibroblast migration in 3D collagen: the native fibrillar structures within 3D ECMs promote integrin activation and clustering, which necessitates physical force for adhesion disengagement. Altogether, these two concepts illustrate how cellular structures, movements, and overall migration are regulated by the local physical attributes of 3D ECM.

The discovery that adhesion-scale differences in fibre stiffness were drastically different in heterogeneous matrices of mixed single- and parallel-bundled collagen fibrils suggests that measurements of the average stiffness of a 3D gel do not indicate the local stiffness a cell may ‘feel’ at the fibril/cell adhesion scale. Single collagen fibril stiffness can be in the MPa range when measured along its length^33^,^34^,^35^. However, this stiffness is much lower when measured perpendicular to the long axis, which commonly occurs with randomly aligned collagen gels, and which we suggest can account for the several-magnitude differences in mechanical stiffness. Previous studies characterizing collagen hydrogel stiffness have mostly utilized bulk rheological measurements that are above the cellular scale, and have often applied shear force to extrapolate gel stiffness^25^. Our findings suggest that for heterogeneous matrices, the local micron-level differences of individual fibril stiffness are likely to be an important factor in regulating individual cell adhesion-level mechanical responses. Other local changes in fibril microstructure such as extent of interfibril branching have been shown to effect the 3D vascularization of endothelial cells and crosslinking via lysyl oxidase can alter cancer cell migration, indicating that other micron-scale alterations to ECM stiffness can alter cellular events^36^,^37^,^38^.

In this study, we do not establish definitively whether the stiffness or the increased fibril surface area has greater responsibility for the increased adhesion maturation and stabilization we observe in FB matrices. However, we found that adhesion alignment along a fibril is particularly strongly associated with adhesion stabilization in FB gels; in fact, collagen fibrils have MPa stiffness along their long axis, but show reduced stiffness when bound from the side^35^. This finding may complement the findings of Kubow *et al.*^24^ that fibril alignment can dictate adhesion size. Overall fibre size has been reported to play a role in 3D migration^39^. However, data from 2D studies, where adhesion size is not restricted, indicates that ECM stiffness is the major parameter determining adhesion longevity^9^. The local pulling away of adhesions from small fibrils within homogeneous matrices is consistent with active cell probing of the environment to establish a proper foothold, similar to fibroblast probing of highly compliant 2D surfaces^40^. It is likely that similar probing occurs on stiff bundled fibrils before rapid stabilization. Our results indicate that fibroblasts react to fibre stiffness through their ability to stabilize adhesions to promote migration.

Previously, investigations of fibroblasts within 3D matrices have demonstrated differences in cell adhesions and overall cell morphology when compared with 2D flat surfaces or cells seeded on top of collagen gels^41^,^42^,^43^,^44^. Grinnell showed that fibroblasts generate numerous dendritic processes within collagen gels that can be enhanced by the addition of PDGF^42^, while others have shown a more spindle-like, highly polarized morphology similar to that in 3D cell-derived matrices^44^,^45^. It should be noted that differences in collagen concentration can greatly alter the matrix pore size, which can alter cell morphology^4^. Using a constant concentration of 3 mg ml^−1^ collagen, we find here that the matrix architecture may in fact be the major contributor to fibroblast morphology. The short, thin fibrils that form a rather tightly woven reticulum with small ECM pore size found in our HR condition do not appear to orient cell processes, while a FB matrix consisting of the bundled, thick long fibrils with larger pore sizes can promote contact guidance with multiple pseudopods extending along fibrils during migration (Fig. 2f and Supplementary Fig. 1d). In fact, fibroblasts seeded on top of thick collagen gels fail to invade into HR ECMs (<20 vertical microns in 72 h), yet can traverse 300 vertical microns in FB4 ECMs within 24 h (data not shown), further implicating matrix architecture in regulating migration. Data from Wolf *et al.*^26^ as well as our own suggest that these two ECM architectures mimic those found *in vivo*, with each presenting different physical obstacles to efficient cell migration.

Our data indicate that fibroblast adhesions within 3D collagen are similar to those found in 3D cell-derived matrices except for the absence of α5 integrin^45^, yet adhesion molecular composition was not sensitive to the differences in ECM architecture we explored here. However, ECM architecture-dependent changes in zyxin turnover, adhesion lifetime and the proportion of mature to retracting adhesions do occur, indicating that the local microenvironment can regulate 3D adhesion dynamics and cell migration in 3D, which may result from subtle shifts in adhesion/ECM coupling. As summarized in Fig. 8d,e, increased ECM stiffness affects the entire adhesion population in several ways: (1) adhesion maturation is increased; (2) the population of mature adhesions undergoing retraction at the leading edge is reduced, reflecting enhanced local ECM/adhesion coupling; and (3) the population of highly stable adhesions increases. The reduced mobility of zyxin points to more efficient adhesion/ECM coupling with reduced adhesion retraction.

ECM-dependent control of cellular dynamics has been explored in 2D cell migration. Lamellar actin flow rate shows an inverse relationship to adhesion lifetime as ligand density increases^19^. Conversely, increasing 2D substrate compliance increases the frictional slipping between integrins and the underlying ECM^13^, which may be analogous to increased adhesion retraction in softer matrices in our study. Hence, several ECM-dependent regulators in 2D correlate with adhesion lifetime and dynamics, as we observed in 3D environments. Interestingly, even though we classified the majority of 3D adhesions as intermediate, it is the small nascent, retracting and highly stable adhesion populations that determine the cellular response and become rate limiting. Recent evidence suggests these types of population-based shifts can occur at the molecular level within adhesions^46^.

So how does this local regulation of adhesions by fibre stiffness promote cell migration? Our findings suggest increased fibre stiffness can withstand the repetitive contractile pulling at adhesion sites, allowing adhesion/ECM coupling that supports adhesion stability. Stabilizing leading edge adhesions promotes further protrusion, as we previously showed for migration on 1D fibre-like micropatterns^20^. These adhesions provide an anterior foothold and together with the stable adhesions surrounding extending pseudopods, create a mechanical axis to promote cell migration. Thus, extracellular factors can indirectly affect downstream mechanisms. The different ECM-dependent shifts in adhesion populations in low-contractility (5 μM blebbistatin) and ligand/integrin interaction experiments suggest a required force balance dictated or set by the ECM, which could be conceptualized as shifting the adhesion population above or below a critical ‘balance threshold’ to regulate migration (Fig. 8e). We further elaborate and present a model for the control of adhesions in 3D as supplemental information (Supplementary Fig. 8).

Previous investigations have shown a requirement for contractile force in 3D cell motility^4^,^23^,^42^,^47^,^48^,^49^, which for cancer cells could be pore size dependent. Here we illustrate that the loss of contractility leads to similar decreases (∼50%) in fibroblast motility regardless of the 3D matrix pore size; this surprising finding is likely due to the high degree of activated integrins. The associated elevated level of integrin binding may represent a significant rate-limiting step for fibroblast migration but not for certain cancers cells due to the differential mechanical interactions of fibroblasts within collagen gels. A discrepancy between the ECMs was only observed under low-contractility conditions (5 μM blebbistatin) and only for HR ECMs; in this condition, intrinsic cellular contractility appeared to be unbalanced with respect to the low ECM stiffness. We hypothesize that in this situation, cells likely contracted or pulled harder than the highly elastic fibres could withstand before ECM ligand interactions were disrupted. Focal adhesions are known to be sensitive to force, requiring it for their stabilization; however, continually increasing force can initiate adhesion disassembly and retraction^9^,^50^. Reducing contractility with 5 μM blebbistatin allowed partial stabilization of adhesions and enhanced, rather than reduced, migration rates in HR collagen gels. This instability in HR ECMs could also be alleviated by increasing ligand interaction via FN treatment or increasing integrin activation using two different approaches. By partially reducing contractility, cellular force became balanced with the low ECM stiffness. However, a complete loss of contractility reduced the ability of cells to disengage clustered, activated integrins.

The differential regulation of 2D and 3D cell migration by cytoskeletal contractility has been a conundrum attributed to ECM-dependent confinement^4^,^20^,^47^. While this concept appears correct for ECMs with limited pore size (HR condition in this study), increasing matrix pore size should reduce this effect. Instead, our data suggest that for fibroblasts, a key process is disengaging clustered, activated integrins. Although integrin activation is a force-independent event, mechanical force is a prerequisite for integrin clustering and strengthening 2D adhesions^51^. Our findings that integrin clustering can occur along collagen fibrils without myosin II contractility suggests the nanoscale geometry of ECM binding sites alone may promote integrin clustering. In fact, integrin activation negated the contractility-independent acceleration of 2D migration, so this discrepancy depending on dimensionality may be due to increased drag on the membrane from enhanced integrin ligation.

While determining the roles of local 3D microenvironments in cell migration is challenging, our findings reveal the importance of local cell-matrix dynamics at the subcellular cell adhesion scale. Here a constant balancing of local internal and external mechanical signals culminates in global differences regulating the efficiency of 3D cell migration. Because *in vivo* ECMs with diverse architectures can be found juxtaposed to each other, or even change with age or disease progression, our results highlight the importance of understanding how these physical aspects of the ECM can alter cellular mechanics. In the future it will be pertinent to understand how other cell types, including cancer cells, diverge from this mesenchymal fibroblast blueprint of 3D mechanotransduction.

## Methods

### Cells culture and transfection

Human foreskin fibroblasts (HFF) were a kind gift from Susan Yamada (NIDCR/NIH) and were derived from human foreskin tissue samples provided by the Cooperative Human Tissue Network (funded by the National Cancer Institute). HFFs were cultured in phenol red-free DMEM (Hyclone) containing 10% fetal bovine serum (Hyclone), 100 U ml^−1^ penicillin/streptomycin (GIBCO), and 2 mM L-glutamine (GIBCO) at 37 °C with 10% CO_2_. eGFP-zxyin null MEFs were a gift from Mary Beckerle (University of Utah) and were maintained at 37 °C and 5% CO_2_ in DMEM supplemented with 10% fetal bovine serum (Hyclone).

### Plasmids and transfections

pmApple-paxillin was from Mike Davidson (Florida State University). pEYFP-paxillin was generated by subcloning the sequence encoding human paxillin into the HindIII–XbaI sites of pEYFP-C1 (Takara Bio Inc.) that contained a modified multiple cloning site. pEGFP-tensin 1 was described previously^52^. pEYFP-talin was generated by subcloning the sequence encoding human talin (from Richard Hynes) into the Not1-EcoR1 sites of pEYFP-NBC1 that contained a modified multiple cloning site. EGFP-lifeact was purchased from Ibidi. Plasmids were transfected into fibroblasts by electroporation using a Bio-Rad Gene Pulsar TM at 170 V, 960 μFd with external capacitance and a time constant of 17–22 μs in 0.4-cm gap cuvettes.

### Reagents

Sources were: dimethyl sulfoxide (DMSO; Sigma), Atto-488 and Atto-647N dyes (Sigma), mouse anti-vinculin (Sigma, 1:200,Cat# V9131-.5ML), rat anti-mouse CD29 (clone: 9EG7; BD Pharmingen,10 μg ml^−1^, Cat# 553715 ), mouse anti-paxillin (BD Pharmingen, 1:75, Cat# 610052), mouse anti-beta 1 integrin (clone TS2/16; Novus Biologicals, 2–10 μg ml^−1^, Cat# NB100-77779), rhodamine phalloidin (Molecular Probes, 1:200, Cat# R415), mouse anti-phosphotyrosine (Upstate 1:100, Cat# 05-3211MG), rabbit anti-mouse collagen type I (Millipore, 1:200, Cat# ABT-123), blebbistatin (–/–; Calbiochem or Cayman Chemicals); rabbit anti-fibronectin R745, rabbit anti-beta 1 integrin (mAb13), and rabbit anti-talin (4392) were generated in our laboratory and used at 10 μg ml^−1^ unless otherwise specified.

### Activation of glass imaging chambers

Glass-bottomed dishes (MatTek Corp., #1.5 thickness coverglass, 20-mm imaging area) were acid-washed with 68% nitric acid (Fisher Scientific) for 25 min, rinsed under a continuous flow of dH_2_O for 1 h, treated with 200 mM NaOH for 15 min, rinsed twice with dH_2_O, then dried under forced air and kept covered until needed. Triethoxysilylbutraldehyde (Gelest Inc.) was diluted to 2% in 100% ethanol then added to glass surfaces and incubated for 5 min. Silane solution was aspirated then rinsed two times with 100% ethanol and once with dH_2_O. Surfaces were then blown dry with forced air and cured at 65 °C for 2 h, and finally stored desiccated at 4 °C.

### PVA blocking of glass coverslips

To locally deter collagen attachment to the center of the 20-mm imaging area, custom-made O-rings (inner diameter of 10 mm and outer diameter of 16 mm) were mechanically punched from Press-to-Seal silicone sheets (Invitrogen). O-rings were centred within the 20-mm area and firmly pressed in place, creating a watertight seal. A marker was used on the underside of the dish to indicate the inner edge of the washer for reference while imaging. PVA (molecular weight 98,000; 98% hydrolysed; Sigma-Aldrich) was diluted in H_2_O to a 6.5% stock solution. This mixture was solubilized at 90 °C in a water bath and was immediately 0.2-μm filtered to remove impurities.1,124 μl 2N HCl was added to 8,876 μl of the PVA solution (∼6% PVA). 200–400 μl of the PVA solution was added to the center of each washer and incubated in a covered humid container for 40 min. The solution was gently aspirated from the surface and washed three times with dH_2_O. The aldehyde-hydroxyl bonds were reduced through treatment with 800 μg ml^−1^ NaBH_4_ in 200 mM ethanolamine buffer for 8 min. After rinsing three times with dH_2_O, the silicone washers were removed and dishes were kept in an enclosed humid environment and used within 48 h. Cells were only imaged within the 10-mm unattached region to rule out boundary effect issues.

### Collagen gel formation

Rat tail monomeric collagen was a kind gift from Greg Kitten and was prepared similar to Chandrakasan *et al.*^53^. Briefly, rat tail tendons were dissected out with special care given to remove fragments of bone, cartilage, blood vessels and even the tendon sheaths, all of which can contribute ‘contaminating’ collagens (II, III, IV and so on) and other ECM components (fibronectin, proteoglycans) which may cause lot-to-lot variations. Tendon fibres were then suspended in 0.5 M acetic acid and stirred at 4 °C for 48 h. The resulting solution of solubilized collagen was filtred through several layers of gauze and centrifuged at 14,000 × *g* for 1 h. The supernatant was collected and then dialysed against three changes of acetic acid (0.02 M) at 4 °C over a three-day period. Protein concentration was determined by comparing pre and post weights following lyophilization of five to six tubes containing 1 ml of solution and was confirmed with a Sircol collagen assay kit (Biocolor).

A stock collagen I solution was generated on ice by mixing rat tail collagen I (6.03 mg ml^−1^) with 10 × DMEM (Sigma) and 10 × reconstitution buffer (200 mM HEPES, 262 mM NaHCO_3_) in a 10:1:1 ratio. The pH was then adjusted to 7.4 with 1N NaOH. PBS^++^(PBS containing both calcium and magnesium) and cells (5.0 × 10^5^ cells per ml collagen) were finally added to bring the final gel concentration to 3.0 mg ml^−1^. One hundred and fifty microlitres of the gel was added to a 35-mm MatTek dish (20 mm glass, #1.5 thickness). HR Gels were polymerized at 37 °C in a tissue culture incubator (10% CO_2_). LR and FB16 gels were polymerized on an Echotherm chilling/heating dry bath (Torrey Pines Scientific) at 21 and 16 °C, respectively. FB4 gels were polymerized at 4 °C without cells in a refrigerator after sealing the dishes with parafilm. Gel polymerization times varied between conditions and were approximately 15, 20, 45 min and overnight for HR, LR, FB16 and FB4 gels, respectively, after which all gels were allowed to reach room temperature (∼22 °C) for 10 min (30 min for the 4 °C condition) before medium or PBS^++^ at the same temperature was added. Collagen turbidity assays (data not shown) were performed to verify that the collagen gels were completely polymerized using a PerkinElmer LS 55 fluorescence spectrometer (UK) with excitation and emission set to 600 and slits set to 2.5 nm. After FB4 gel polymerization, cells were added to the 3D lattice and allowed to invade for 48 h before imaging. The polymerization temperature did not affect cell viability or function, as cell migration was normal, and few if any cells showed signs of apoptosis in all conditions. For AFM experiments, gels were polymerized the day before measurements, then PBS^++^ containing 100 U ml^−1^ penicillin/streptomycin was added as described above, sealed with Parafilm, and maintained at 4 °C until use (within 48 h).

### Fluorescent labelling of collagen

To fluorescently label collagen gels, the method of Ghersi *et al.*^54^ was modified. Briefly, 5 ml of a 3 mg ml^−1^ collagen I solution was polymerized at room temperature. After gelation, the gel was incubated with 50 mM borate buffer (pH 9.0) for 10 min. This was aspirated and replaced by 5 ml of a NHS-ester dye solution in the same buffer and incubated at room temperature in the dark for 1 h. The concentration of dye (diluted in DMSO) varied and was based on the dye’s molar-excess calculated by the company. The dye solution was aspirated, and any remaining NHS-esters were quenched with 10 ml of 50 mM TRIS buffer (pH 7.5) for 10 min. The gel was then rinsed 6–10 times with PBS^++^ over several hours. Gels were then acidified in 200 mM HCl and mixed until the gel was completely solubilized. The collagen solution was then dialysed against 20 mM glacial acetic acid (Fisher Scientific) for 4 h at a 1:1,000 ratio. Collagen concentration was measured using a Sircol collagen assay kit. A fluorescent collagen stock solution was created and mixed in bulk (6 ml at a time) for consistency. About 2–4% of the unlabelled collagen I solution was removed and replaced with the same amount of labelled collagen (calculated based on protein weight). The final collagen I solution concentration was then calculated and used for all experiments.

### Immunofluorescence staining

All fixation and permeabilization steps were performed at 37 °C. Cells were permeabilized and fixed in 3% paraformaldehyde (Electron Microscopy Sciences), 0.5% Triton X-100 (Sigma), with 10 μg ml^−1^ nonfluorescent phalloidin (Invitrogen) in cytoskeletal buffer (CBS; 10 mM MES, 138 mM KCl, 2 mM EGTA, 3 mM MgCl_2_ plus 5% sucrose) for 90 s, post-fixed in 4% paraformaldehyde in CBS for 15 min. Cells were rinsed 3X in PBS^++^ and permeabilized with 0.5% Triton X-100 in CBS for 5 min. Cells were rinsed 5 × over 40 min with PHEM+glycine (60 mM PIPES, 2 mM HEPES, 10 mM EGTA, 2 mM MgCl_2_, 100 mM glycine, pH 6.9). Non-specific sites were blocked with 20% donkey serum (Jackson ImmunoResearch Laboratories), together with M.O.M. reagent (Vector Laboratories), in PHEM+glycine buffer for 1 h. Cells were rinsed three times with PHEM+glycine over 30 min. Primary and secondary antibodies were diluted in PHEM+glycine with 10% donkey serum and incubated for 45 and 25 min, respectively. Secondary antibodies were either from Jackson ImmunoResearch Laboratories or Molecular Probes.

### 3D cell adhesion processing and stability calculations

To reduce the background of collagen image stacks, a rolling ball subtraction (radius=5 pixels) was applied before deconvolution. All 3D Z-stacks were then deconvolved using an adaptive point spread function with AutoQuant (Media Cybernetics, Bethesda MD). Spherical aberration was corrected and the distance from coverslip was taken into consideration during processing. The number of iterations was sample dependent. Following deconvolution, adhesion image Z-stacks were filtered using 2 × 2 low-pass filter and a 9 × 9 unsharpen mask (scaling=0.5). From these images stacks, an average projected image was subtracted from a maximum projected image for each time point to enhance adhesion structures. For deconvolved collagen Z-stacks, no other filtering was applied. The average and max projected images of these Z-stacks were added together and used for analysis and images.

### 3D adhesion tracking

Adhesions and/or fiduciary marks in the collagen ECM were tracked using Speckle TrackerJ, an ImageJ plugin originally developed for speckle microscopy^31^. From this analysis, adhesion lifetime and movement were calculated and vector and adhesion track images created. Briefly, the diffusing _NCC model was used to track adhesions/spots of 5 pixels or larger (∼1 micron). Intensity threshold (on a 16-bit scale) varied depending on the cell. A minimum track separation was set between 3–8 pixels. The minimum duration was set to three frames (60 s) to exclude noise spikes being counted. Link frame distance was set to three frames. Search size was set to three. All adhesions were auto-tracked by the software and then individually verified and corrected by A.D.D.

### FRAP analysis

FRAP kinetics were analysed similar to Humphries *et al.*^55^. Briefly, each FRAP time series was adjusted for photobleaching using ImageJ software (NIH, Bethesda, MD). To calculate *t*_1/2_ times, fractional fluorescence was plotted based on calculations from Snapp *et al.*^56^. 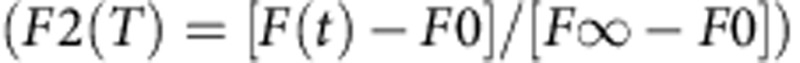. FRAP curves were fit to single exponentials using Prizm4 by Graphpad. *k*_off_ rate was analysed similar to Lele *et al.*^28^.

### Dermal explants and mouse back skin preparations

Dermal explants were isolated from 12-week-old pregnant female ICR mouse ears (mice obtained from Harlan Laboratories) and were prepared by separating the dorsal and ventral surfaces as described by Lammermann *et al.*^57^ Back skin was isolated from E17.5 ICR mouse embryos (with morning of plug discovery counted as E0.5; also from Harlan). All mice were housed, bred and killed according to an approved NIDCR animal study protocol. Sample were not fixed or permeabilized. Nonspecific protein-interaction sites were blocked with 20% donkey serum (Jackson Immuno-Research Laboratories) together with mouse on mouse reagent (M.O.M.; Vector Laboratories) in PHEM buffer for 1 h. Primary and secondary antibody Alexa-fluor 488 donkey anti-rabbit, Jackson Labs) were mixed with 10% donkey serum in PHEM buffer and incubated for 1 h with 5 washes of PHEM buffer between labelling steps. Imaging was carried out using a LSM 510 NLO META confocal microscope (Carl Zeiss Inc.) equipped with a Apochromat × 63 1.2 NA water objective and a 488-nm Argon laser was used to provide excitation.

### Traction force microscopy

Traction force microscopy was performed as described previously^40^. Briefly, cells transfected with M-apple-N1 were plated on polyacrylamide gels that had a stiffness of 4.3 kPa, embedded with 655 fluorescent microspheres (Life Technologies), and images acquired before and after release of cells with 10% SDS. Images of deformed and relaxed substrates were aligned using MetaMorph software, and movement of the microspheres was quantified with the ImageJ PIV plugin created by Tseng *et al.*^58^.

### Porosity and fibril calculations

To calculate porosity, 2% Atto-488-labelled rat–tail collagen was polymerized and the different permissive temperatures and imaged on a spinning disk confocal microscope with a × 60 objective. Twenty-micrometre thick Z-stacks were imaged every 0.2 microns beginning 5 μm above the coverslip surface. These Z-stacks were then deconvolved with Autoquant software and then subdivided into 4 μm Z-stacks (20 frames), max projected to a single image, and further filtered using a custom Gaussian 5 × 5 kernel, background flattening (pixel width=15), and then a low sharpening (3 × 3) filter in MetaMorph. From these filtered images a thresholding mask was set to the average pixel intensity of the image and grayscales below this value were subtracted from the image. From these images an inverted threshold (dark threshold) was used to calculate the per cent area that was not considered a fibre and used as a per cent porosity measurement. For collagen fibril analysis, line segments were drawn across the widths and lengths of fibrils found in calibrated SIM images for HR and FB16 ECMs using MetaMorph software.

### AFM force volume measurement

The collagen gels were measured by AFM, using a Bioscope Catalyst device (Bruker Santa Barbara, CA) attached to an inverted optical microscope (IX71, Olympus, Japan) in a similar manner as described previously^27^,^59^,^60^,^61^. Briefly, the gels were probed under PBS^++^ with a V-shaped cantilever (conical tipped, nominal *k*=0.01 N m^−1^; Bruker, Santa Barbara, CA), whose spring constant was precalibrated by the thermal fluctuations method in air. The relationship between photodiode signal and cantilever deflection was computed from the slope of the force displacement curve obtained at a bare region of a coverslip or cell culture dish (that is, a hard surface). For each gel, we used force-volume mode in which we acquired multiple force-displacement (*F*-*z*) curves (where *F*=*k*·*d*, *d* being the deflection of the cantilever) by monitoring *F* and *z* while the piezo translator was ramped forward and backward at constant speed (for example, 8 μm amplitude, 1 Hz and ∼1 μm of indentation, much less than the tip height of 2.5 to 8.0 μm) as it moved across an area defined as 32 × 32 μm^2^ or 64 × 64 μm^2^. Each experimental *F*-*z* curve is fitted to the conical indenter Sneddon model:




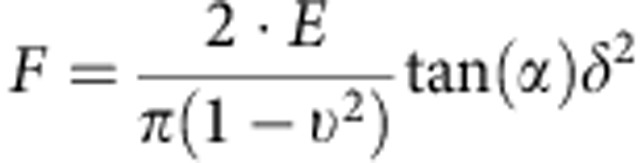




where *E* is the Young’s modulus, *ν* is the Poisson’s ratio, α=18° is the indenter half-angle and *δ* is the indentation depth^48^. The parameter *ν* is assumed to be the instantaneous Poisson’s ratio of incompressible composites, which is 0.5 (ref. 62), as the collagen gels in aqueous environment are idealized to behave at the high rate of AFM indentation^27^. At slower deformation rates, large non-linear stress/strain or under dehydration, a wide range of Poisson's ratios have been documented for reconstituted collagen ECMs^63^ and native tissues such as cartilage^64^, reflecting the underling fibril microstructure and the complexity of modern materials^62^,^63^,^64^. The value *E* and the tip-gel contact point *z*_0_ were estimated by a least-squares fit of this equation to the *F*-*z* curve recorded for each point in the gel area (for example, 32 μm × 32 μm) using Nanoscope Analysis Software. Shear modulus, *G*, is a very common parameter that is published alongside the Young’s modulus, *E*, and for wet collagen gel idealized as essentially incompressible materials^62^ one estimates that




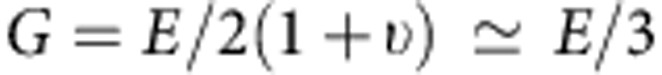




### AFM data analysis

The AFM data were analysed using Nanoscope Analysis Software (Ver. 1.4–1.5, Bruker, Santa Barbara, CA). Considering the viscoelastic nature of collagen, we explored the force volume data (individual force versus displacement curves) by looking at the indentation force^61^. We were able to show the stress stiffening property of collagen by seeing how changes in the indentation force causes differences in the Young’s modulus. When comparing collagen gels of all conditions, we see that the modulus distribution becomes narrower at higher indentation force. We used 10–100% of the baseline to trigger force fit boundaries for acquiring the Young’s modulus values. We only included data points having an *R*^2^ value predominantly between 0.8 and 0.996 and disregarded a small fraction of force curves that did not show a designed trigger force of typically 1 nN.

### Statistics

Prism 4 by GraphPad software was used for all graphs and statistical analysis. One-way analysis of variance, using a Tukey post-test for more than two data sets, and Mann–Whitney *t*-tests, were used to establish significant differences (*P*<0.05). All error bars indicate s.e.m.. In figure legends, *N*=the number of independent experiments, and *n*=the number of data points.

## Additional information

**How to cite this article:** Doyle, A. D. *et al.* Local 3D matrix microenvironment regulates cell migration through spatiotemporal dynamics of contractility-dependent adhesions. *Nat. Commun.* 6:8720 doi: 10.1038/ncomms9720 (2015).

## Supplementary Material

Supplementary Information Supplementary Figures 1-8, Supplementary Discussion, Supplementary Methods and Supplementary References

Supplementary Movie 1 Structured illumination microscopy (SIM) illustrating the strikingly different architectures of homogeneous HR (top) and heterogeneous FB16 (bottom) Atto-488 labeled collagen gels 8-μm Z-stacks.

Supplementary Movie 2 SIM rotational view of a 25-μm thick FB16 Atto-488 labeled collagen gel.

Supplementary Movie 3 SIM rotational view of a 25-μm thick HR Atto-488 labeled collagen gel.

Supplementary Movie 4 Timelapse movie of a fibroblast transfected with EGFP-Lifeact (green) demonstrates contact guidance-based protrusion along bundled collagen fibrils within a FB4 ECM (red). 30 seconds between frames. Total time 45 minutes.

Supplementary Movie 5 Timelapse movie of a demonstrating of adhesion tracking within 3D FB4 collagen gels (red). EYFP-paxillin (green: 1st panel, white: panels 2-5) adhesions are shown with tracks (magenta) and vectors (green) that illustrate the local movements of cellular adhesions during 3D migration. 30 seconds between frames. Total time 33.5 minutes.

Supplementary Movie 6 Timelapse movie of a HFF expressing EYFP-paxillin (green or white) migrating through 3D HR collagen (red). Vectors (right panel) show the retrograde movement of adhesions. Red vectors illustrate adhesions that undergo rapid movement indicative of adhesion retraction. 30 seconds between frames. Total time 48.5 minutes.

Supplementary Movie 7 Timelapse movie of a HFF expressing EYFP-paxillin (green or white) migrating through 3D HR collagen (red). Right panel shows dual adhesion/ECM tracking (green/red vectors, respectively). Over the lifetime of the adhesion, there is relative lack of adhesion/ECM coupling as shown by the divergence of vectors as the adhesion retracts away from the ECM. 30 seconds between frames. Total time 30 minutes.

Supplementary Movie 8 Timelapse movie of a HFF expressing EYFP-paxillin (green or white) migrating through 3D FB16 collagen (red). Right panel shows dual adhesion/ECM tracking (green/red vectors, respectively). Here in a highly stable adhesion, the relative adhesion/ECM coupling is high as demonstrated by the coinciding vector displacements. 30 seconds between frames. Total time 30 minutes.

Supplementary Movie 9 HFF expressing EYFP-paxillin (green or white) migrating through 3D FB16 collagen (red). Cell was treated with 25 μM blebbistatin at the indicated time point. Note the increase in protrusion and reduced cell body translocation occurring after the relaxation of the ECM. Stage was moved during imaging, and then realigned to track cells over long time periods. 5 minutes between frames. Total time 5 hours.

Supplementary Movie 10 HFF expressing EYFP-paxillin (green or white) migrating through 3D FB16 collagen (red). Cell was treated with 25 μM blebbistatin and 1 μg ml^-1^ mAb13 to reduce β1 integrin binding. Inhibitors were added at the indicated time point. Protrusion initially occurs without cell body translocation. However, this is followed shortly by a release of the cell body, which slips through the ECM, rescuing the contractility-deficient condition. Stage was moved during imaging, and then realigned to track cells over long time periods. 5 minutes between frames. Total time 6 hours.
