# Supplementary material for: Local 3D matrix microenvironment regulates cell migration through spatiotemporal dynamics of contractility-dependent adhesions
Source: Nat Commun. 2015 Nov 9;6:8720. doi: 10.1038/ncomms9720 (PMC4643399; doi:10.1038/ncomms9720)
Supplement: Supplementary Information — Supplementary Figures 1-8, Supplementary Discussion, Supplementary Methods and Supplementary References [file ncomms9720-s1.pdf]

Supplementary Figures

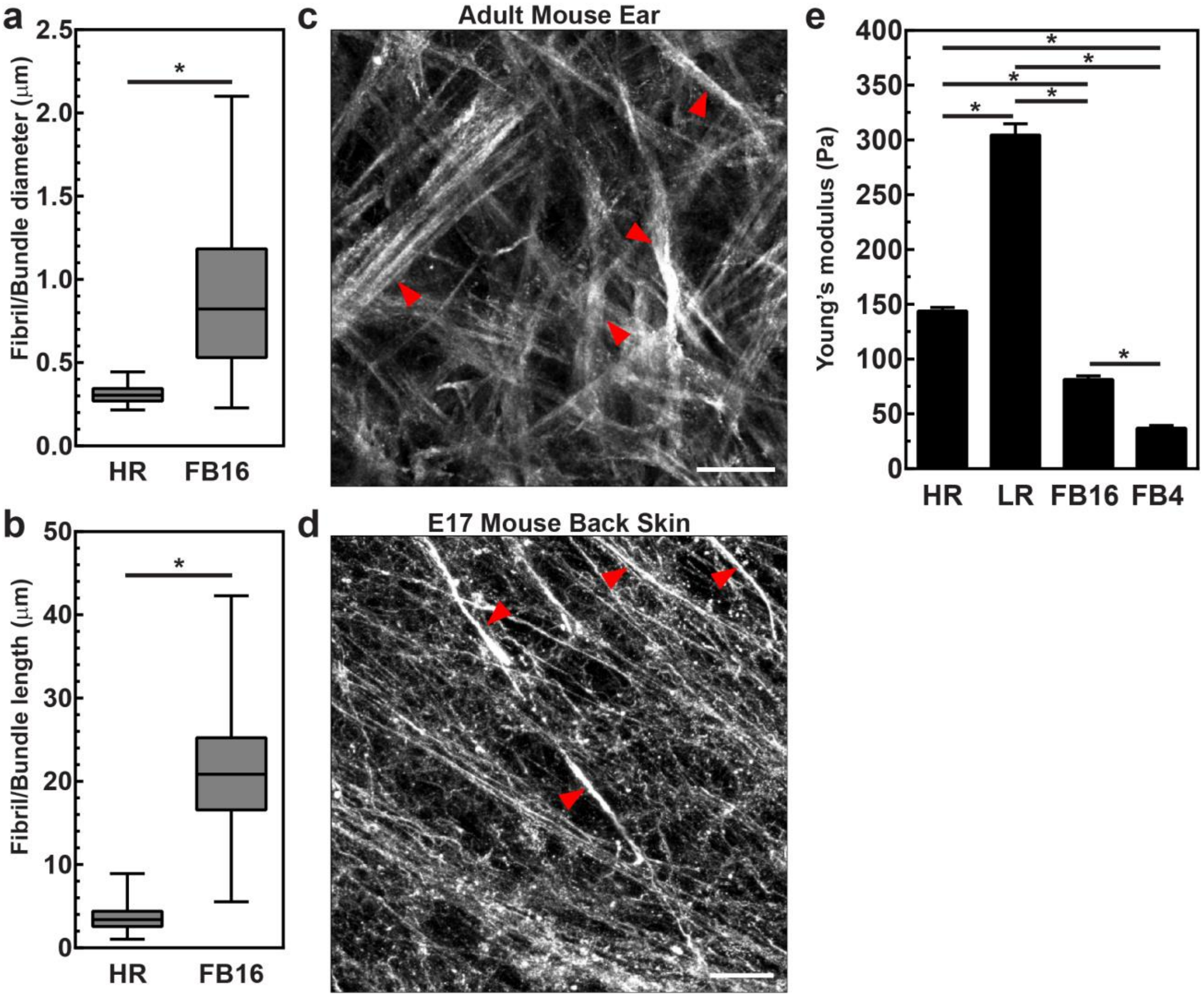

Supplementary Figure 1: Collagen gel fibril sizes in vitro and in vivo. (**a-b**) Box and whisker plots for collagen fibril/bundle diameter (A) and length (B) for HR and FB16 matrices.* *P* < 0.0001, student t-test. N>3, n=100 for both **a** and **b.** Whiskers show minimum and maximum range of data points. (**c-d**) Immunostaining for collagen type I in an adult mouse ear (**c**) and in E17 embryonic mouse back skin (**d**) reveals diverse structural differences in ECM architecture. Red arrowheads indicate bundled fibrils. Scale bars: 20 µm. (**e**) Young's modulus of HR, LR, FB16, and FB4 collagen gels measured using a 38 um beaded AFM cantilever. The reduction in stiffness in FB conditions is due to increased pore size and gel heterogeneity. N>3, n>250. * *P* < 0.0001, ANOVA.

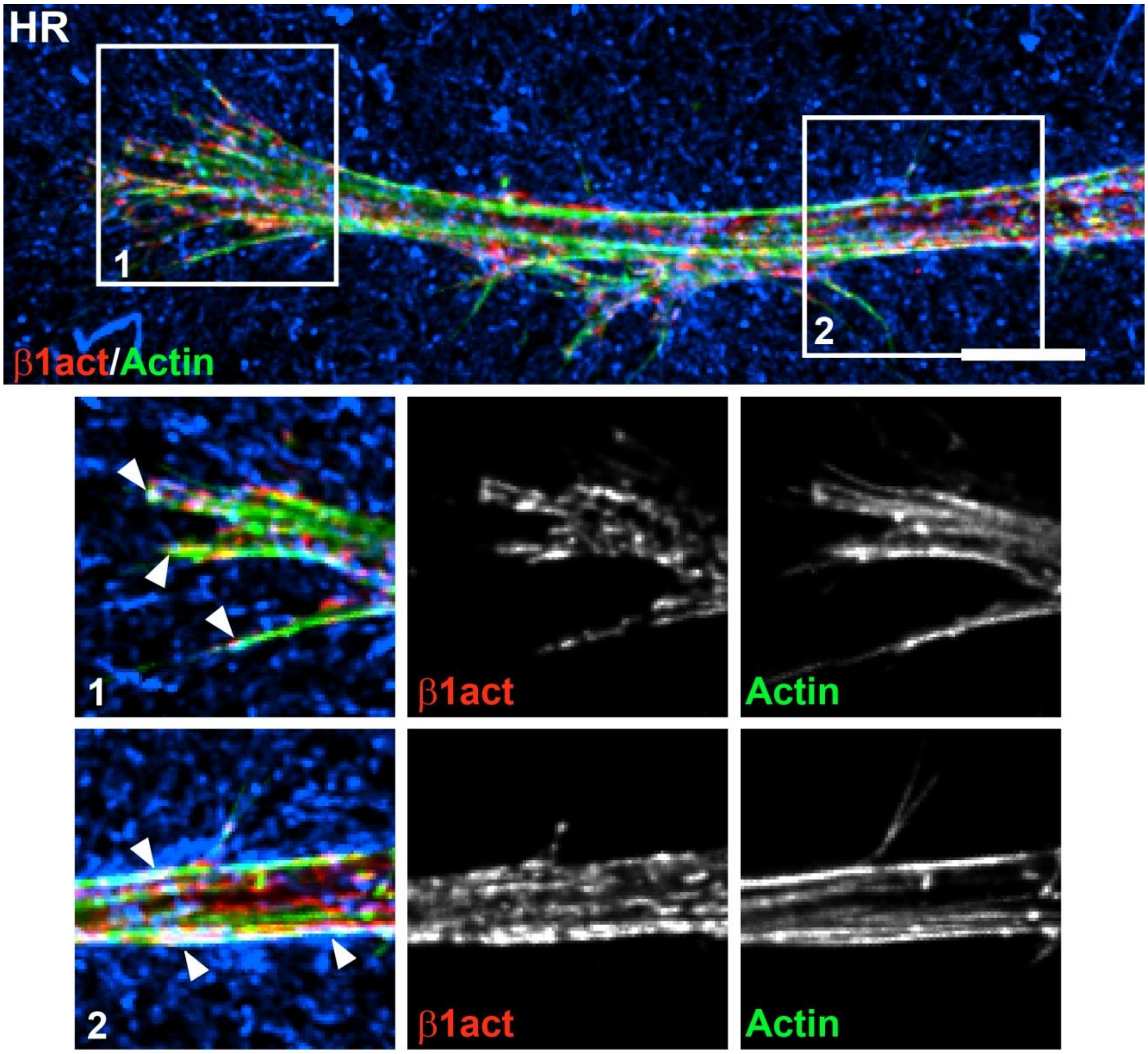

Supplementary Figure 2: Integrin and actin localization in 3D HR ECMs. Full MIP (~15 µm) localizing activated β1 integrin (9EG7: red) and phalloidin staining for F-actin (green) in HR ECMs (blue). Panels 1 and 2 show leading edge and cell body regions, respectively. Arrowheads indicate areas where all three components show alignment. Panels 1 and 2 are partial Z projections of the cell region. Scale bars: 10 µm.

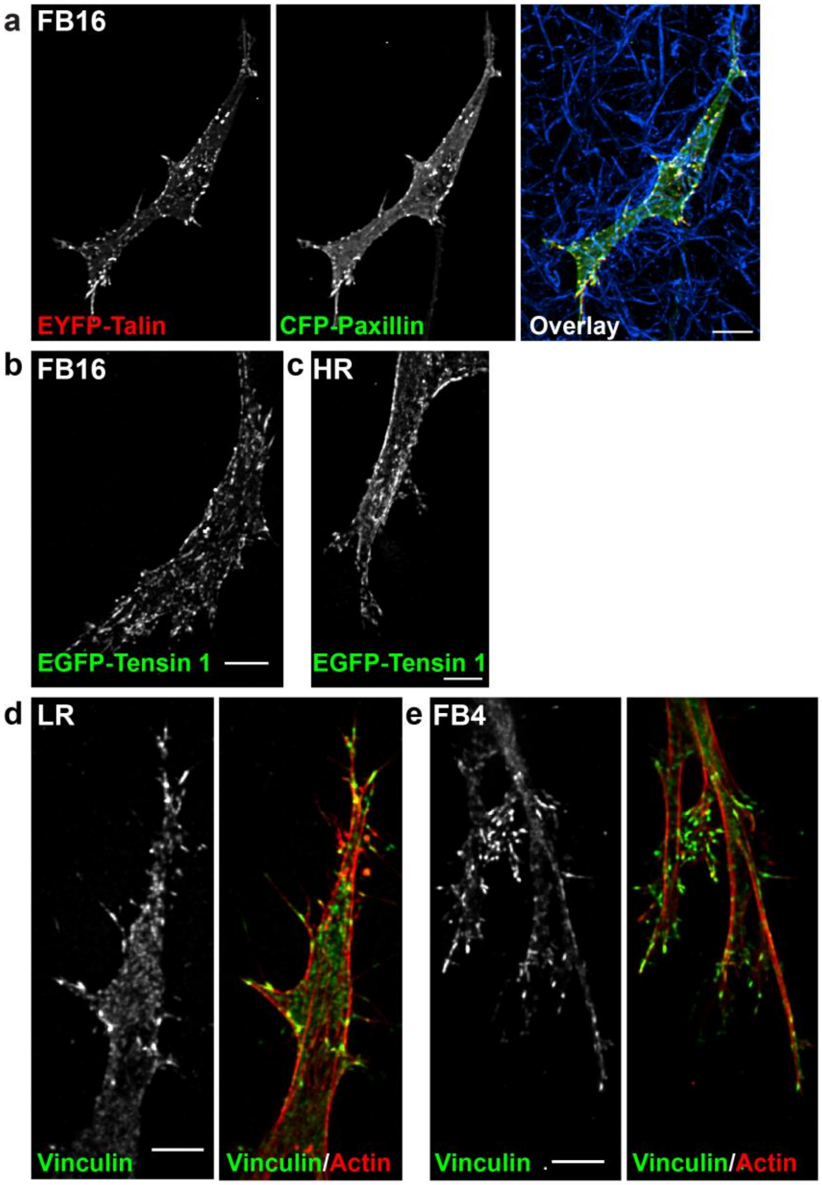

Supplementary Figure 3: Adhesion protein localization in 3D collagen. Immunostaining or expression of fluorescently tagged adhesion proteins in the various ECMs (HR, LR, FB16, FB4). Panels indicate the type of ECM. All images are MIP of Z-stacks. (**a**) EYFP-talin (red) and CFP-paxillin (green) in FB16 ECM (blue, Atto 647N labeled collagen). (**b-c**) EGFP-tensin 1 in FB16 (B) and HR (**c**) ECM. (**d-e**) Vinculin (green) and actin (phalloidin: red) immunostaining in LR (**d**) and FB4 (**e**) ECMs. Scale bars: 10 µm.

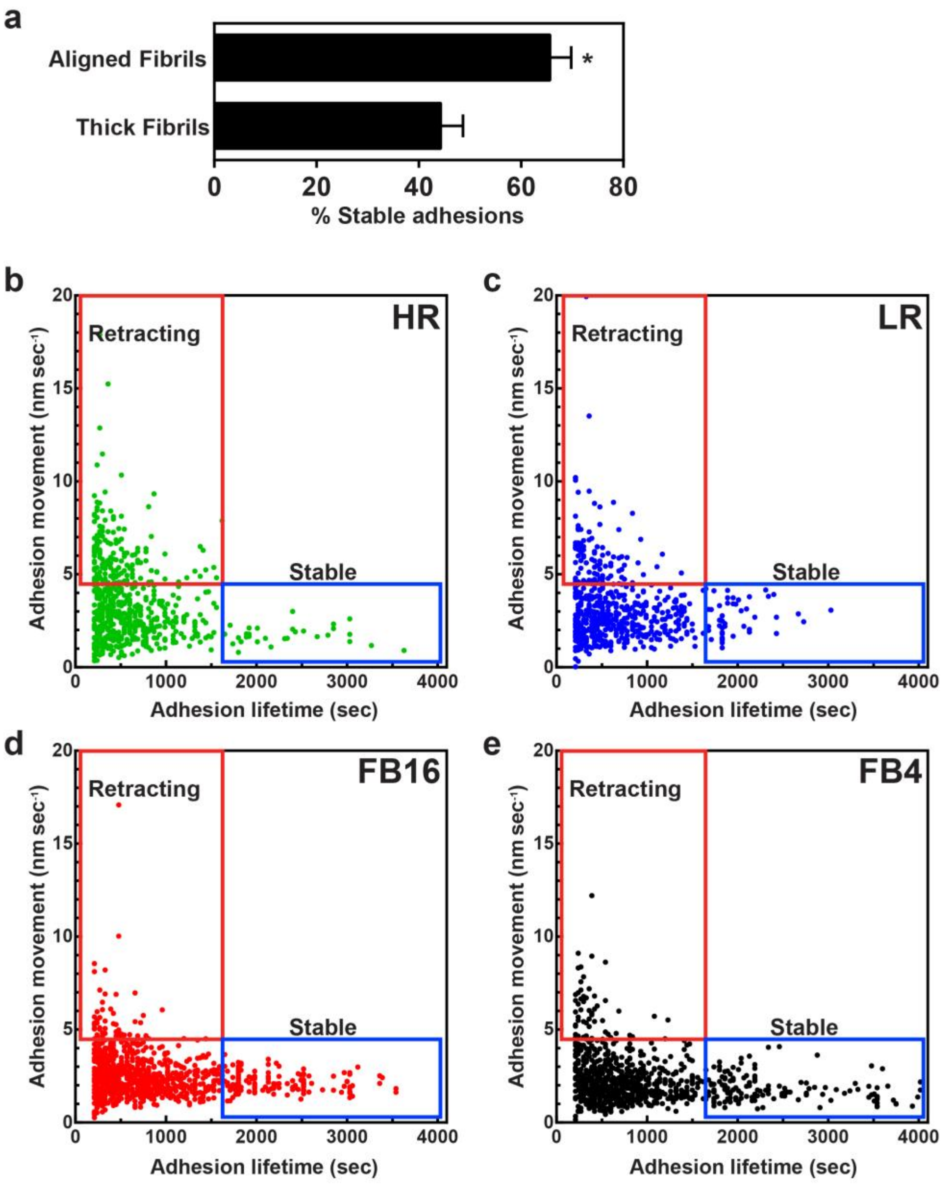

Supplementary Figure 4: Association of stable adhesions and adhesion movement versus adhesion lifetime (**a**) Percent of stable adhesions in FB4 ECMs that are aligned along a collagen fibril or are associated with thick (≥1μm diameter) fibrils.N=6 cells, n>120. *Significantly different; *P*<0.005 Student t-test. (**b-e**) Scatterplot analysis of adhesion movement versus adhesion lifetime for HR (**b**), LR (**c**), FB16 (**d**), and FB4 (**e**) ECMs. Red and blue rectangles highlight the retracting and stable adhesion populations, respectively, and reflect the data illustrated in figure 5F. Panels **a** and **c** are reproduced from figure 5E for comparison. n-values for **b-e** are >500 adhesions and a minimum of 6 cells per condition.

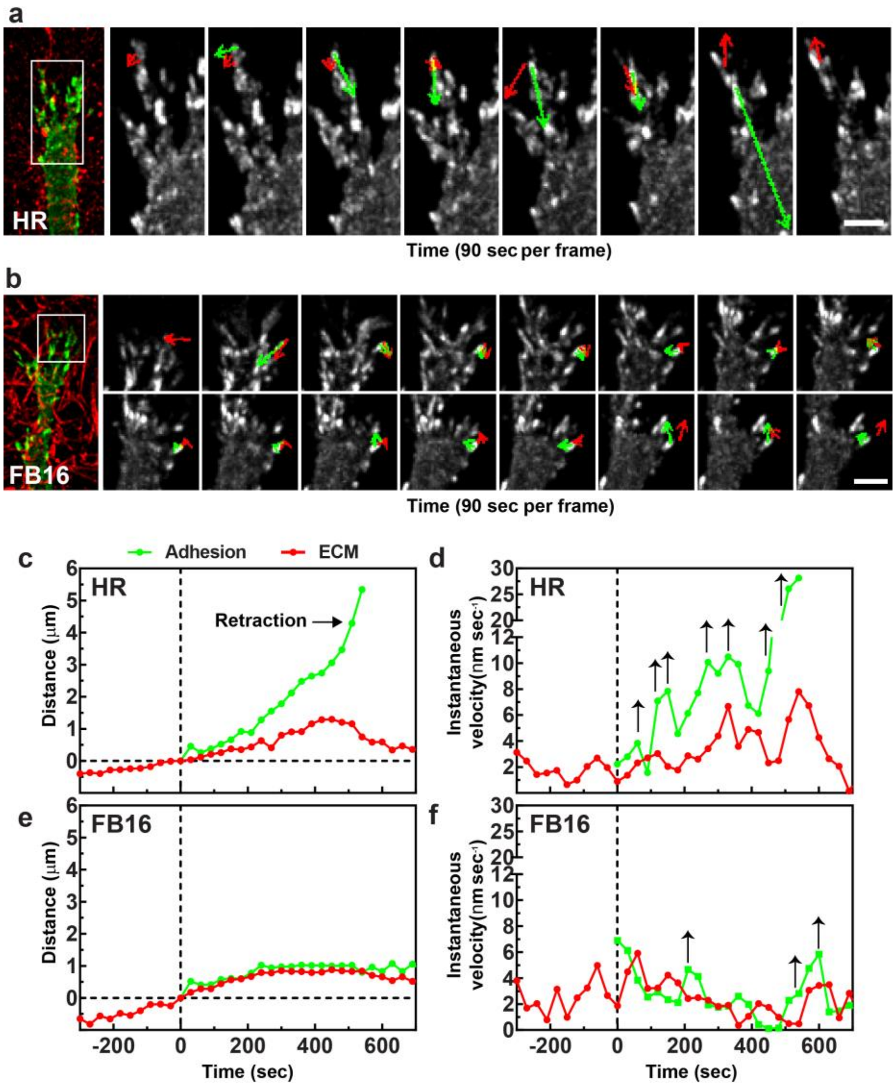

Supplementary Figure 5: Dual tracking of ECM and EYFP-paxillin adhesions. Dual tracking of EYFP-paxillin adhesions (green) and ECM (red) within HR (**a**) and FB16 (**b**) ECMs demonstrates the relative degree of coupling for retracting and stable adhesions, respectively. Vectors depict the direction and magnitude of adhesion (green) and ECM (red) movement. **a** is the same cell shown in Figure 5. (**c** and **e**) Temporal analysis of the distance traveled for the adhesions (green) and ECM (red) indicates a differential coupling to the ECM for retracting (panel **a**) and stable adhesions (panel **b**). (**d** and **f**) Instantaneous velocity of adhesions (green) and ECM (red). Upward arrows indicate where the adhesion is tugging or pulling away from the ECM, which leads to uncoupling and retraction in HR ECMs. Scale bars in **a** and **b**: 4 µm.

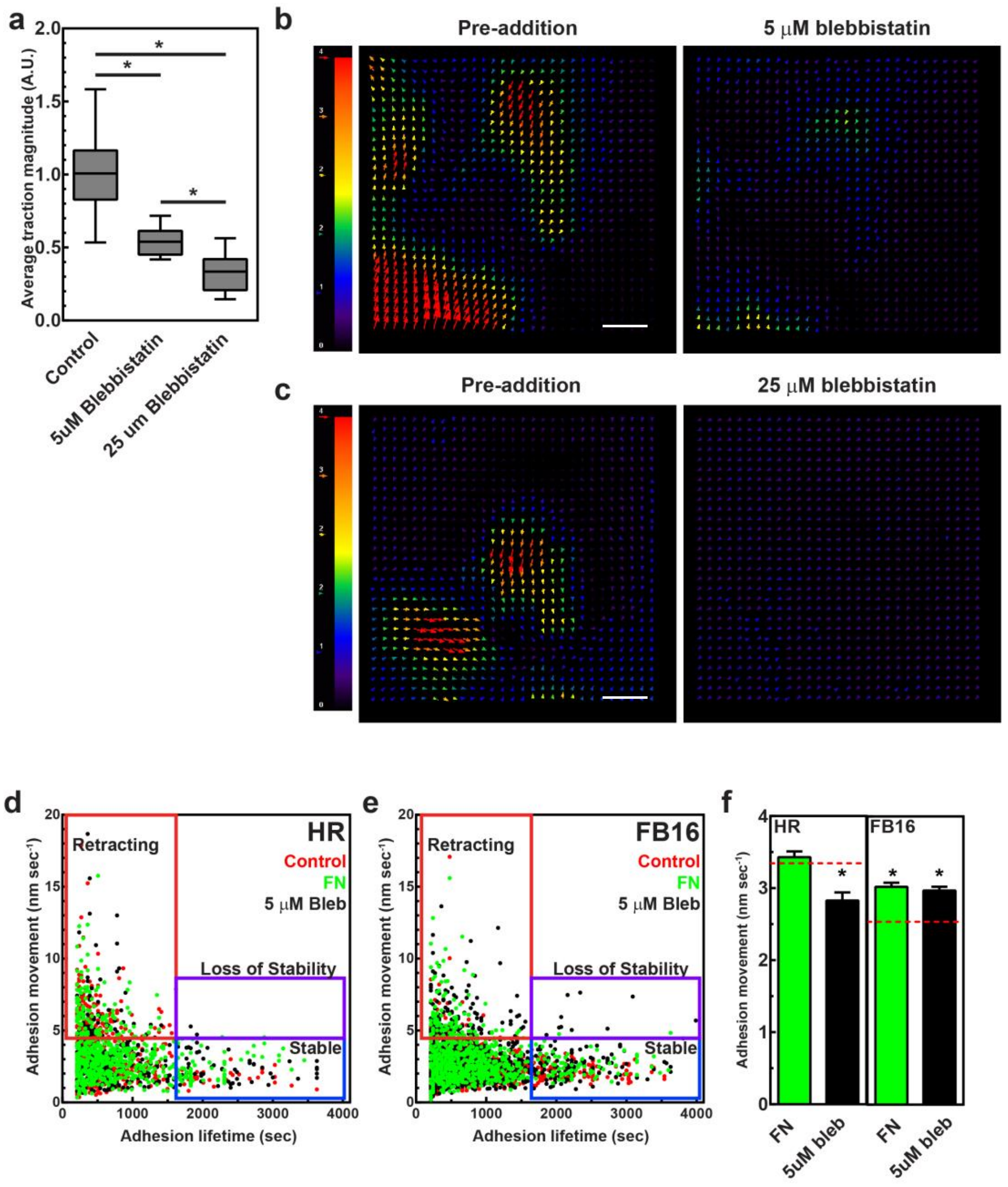

Supplementary Figure 6: Traction force analysis and the change in adhesion stability with FN and 5 µM blebbistatin. Graph (**a**) depicts traction magnitude (A.U.) before (control) and 30 minutes after the addition of either 5 µM or 25 µM blebbistatin. (**b**) Pre (left) and post 5 µM blebbistatin (right) addition. (**c**) Pre (left) and post 25 µM blebbistatin (right) addition. N=3, n≥15.(**d-f**) Scatterplot analysis of adhesion movement versus adhesion lifetime comparing control (red) to fibronectin (green) and 5 µM blebbistatin (black) treatments in HR (**d**) and FB16 (**e**) ECMs. Red and blue rectangles highlight the retracting and stable adhesion populations, respectively. Purple boxes

indicate stable adhesions undergoing retraction (not observed in control conditions). (**f**) Adhesion movement for fibronectin (green) and 5 µM blebbistatin (black) treatments in HR and FB16 ECMs. Red dashed lines indicate control levels. *Significantly different from control condition; *P*<0.05. (**d-f**) Traction force analysis of HFFs plated on 4.3 kPa polyacrylamide gels. *Significantly different; *P*<0.05. Color magnitude scale is in arbitrary units. Scale bar: 10 µm.

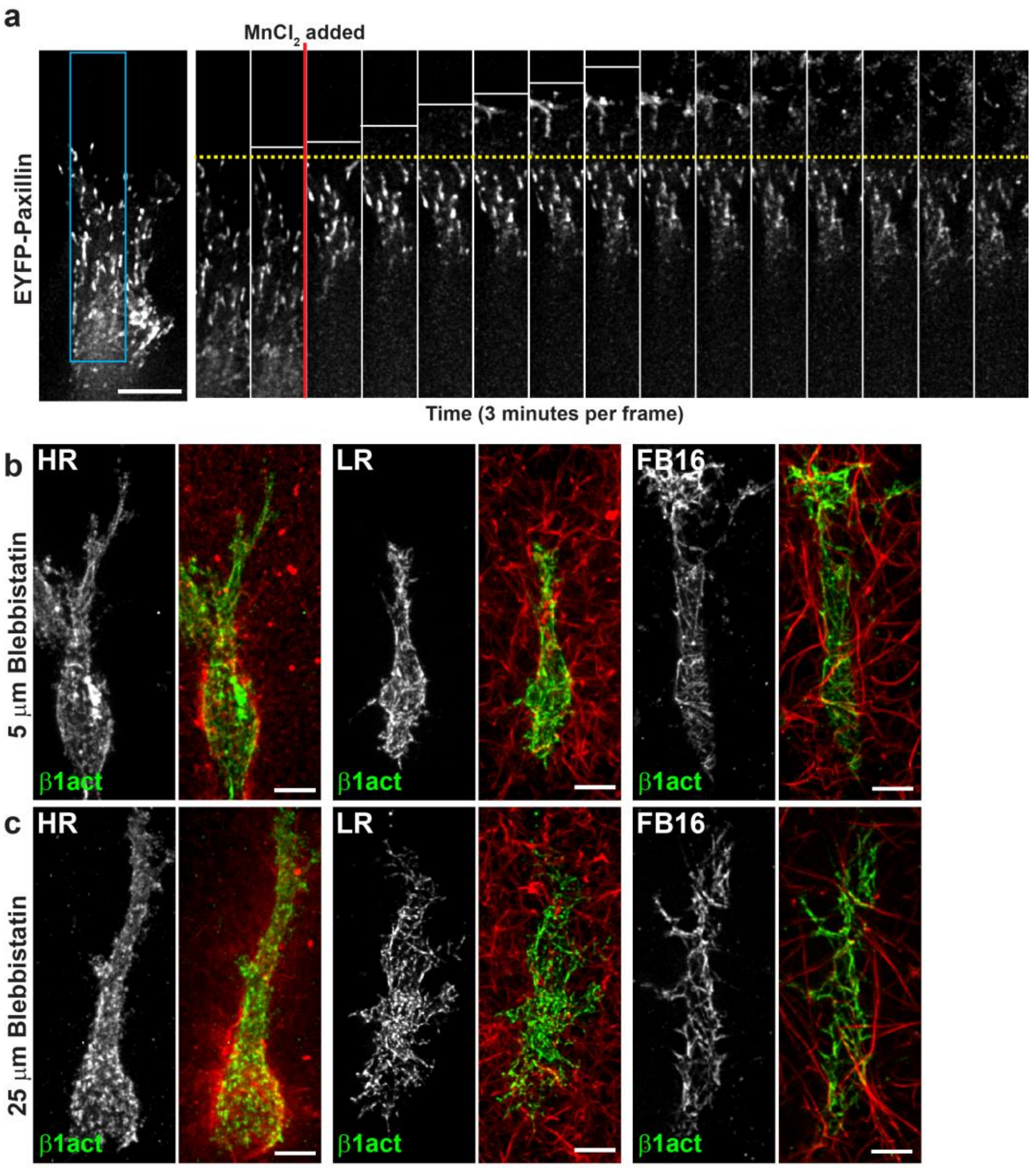

Supplementary Figure 7: Integrin activation stabilizes adhesions and integrin clustering with loss of contractile force. (**a**) Addition of 50 µM $MnCl_2$ stabilizes leading edge adhesions in HR ECMs to promote migration. Time-lapse montage (right) of EYFP-paxillin demonstrates an increase in adhesion stabilization and promotes leading movement after integrin activation by $MnCl_2$. Cyan box indicates the region depicted in the time series. Red line indicates the time of $MnCl_2$ addition. Yellow-dotted line indicates the position of the leading edge in the first frame. White bars mark the leading edge in subsequent frames (moves out of the frame after time point 8). In the 3rd frame, the Z-axis shifts. (**b-c**) Overnight treatment of fibroblasts with 5 µM (**b**) or 25 µM (**c**) blebbistatin

while migrating in HR, LR, or FB4 ECMs (red) results in no significant change in activated β1 integrin (9EG7; green) clustering, indicating that changes in cellular contractile force do not effect integrin clustering. Scale bars: 10 µm.

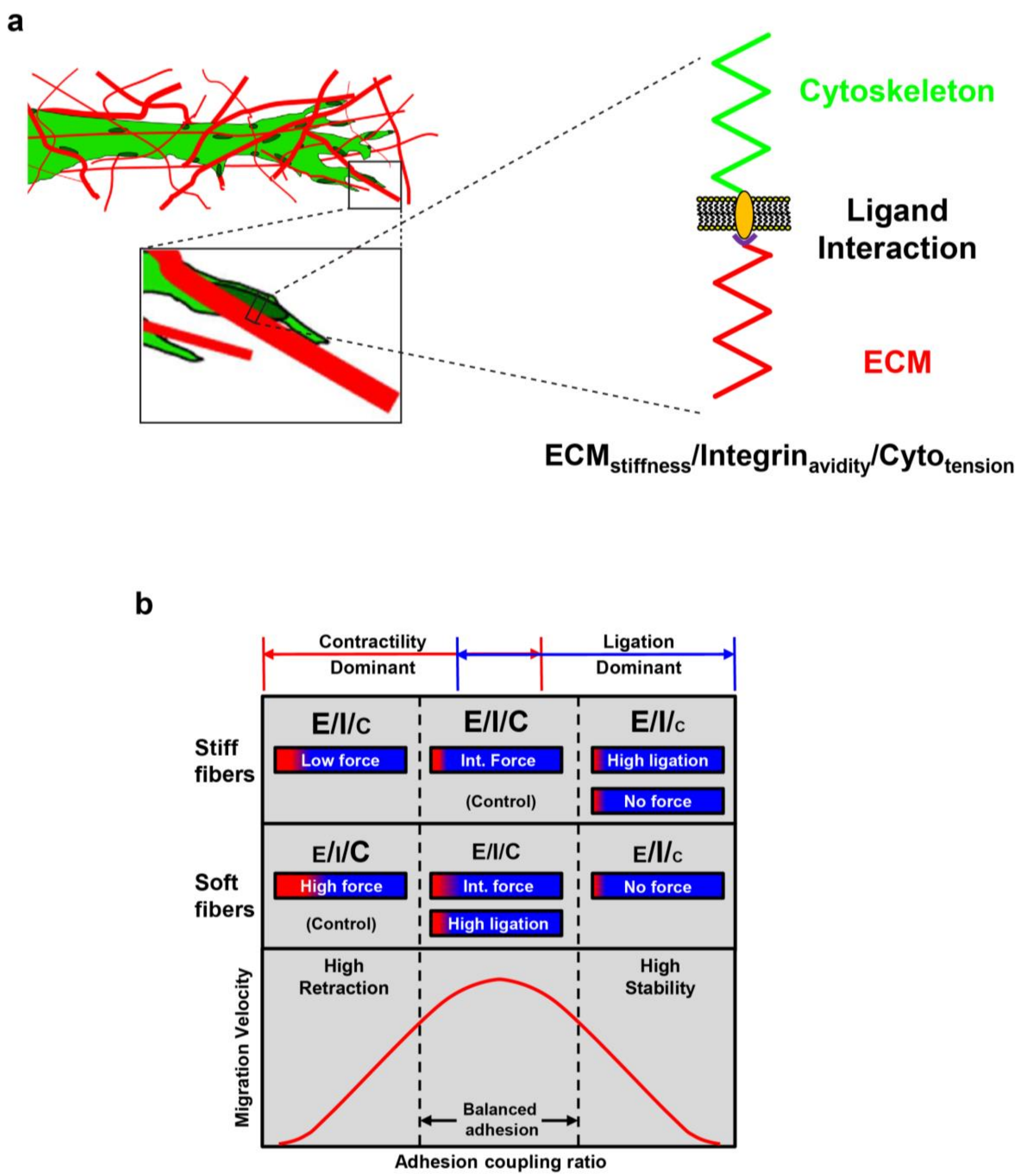

Supplementary Figure 8: Adhesion/ECM coupling in 3D collagen. (**a**) Schematic representation of a cellular interaction with collagen fibrils and the two-spring model originally proposed by Bell[1] and later by Schwarz[2]. Here, the ECM and cytoskeleton act like two springs in series held together by binding of integrins to the ECM. (**b**) Schematic showing how the two-spring model requires an appropriate balance between ECM stiffness (E), integrin avidity for ligand (I), and cytoskeletal tension (C) to regulate cell

migration. The bottom graph depicts how migration velocity is biphasic with respect to adhesion/ECM coupling (A/E). Depicted above the graph are how 3D fiber stiffness shifts the balance of A/E with respect to the two-spring model. The sizes of the lettering for each component (E/I/C) illustrates their relative contribution to the stability of the system. For stiff fibrils, the components are balanced in control conditions (middle portion) with the majority of adhesions maturing and remaining stable during migration due to an intermediate force level. However, for soft fibrils, E and I are low relative to cellular C and results in reduced maturation and increased adhesion retraction, slowing cell migration. The ratio of E/I/C can be balanced for soft fibrils either by reducing cellular forces/contractility or by increasing the avidity of integrins for ligand to partially restore migration velocity; these experimental manipulations have the opposite effect on stiff fibrils. Red/blue bars represent the adhesion population within cells and their current state (red: retracting or nascent; blue: mature or stable).

Supplementary Discussion

Previous theories have defined a framework surrounding the formation and continued stability of adhesions between two interacting surfaces. Bell[1] suggested that adhesion stability is dependent on multiple parameters including the binding and unbinding rates of receptor to ligand (i.e., the “on” and “off” rates) and the forces applied at the adhesion site. Schwarz et al.[2] defined a two-spring model of adhesions as mechanosensors, where the ECM and the cytoskeleton act as two springs in series held together by a focal adhesion (Supplementary Fig. 8a). In this model, a stiffer environment stabilizes an adhesion and reaches equilibrium faster than in a soft environment. Here in this study we find this hypothesis to be correct, where the softer the local microenvironment (external spring) the less stable an adhesion becomes because an equilibrium is never reached, resulting in reduced lifetime, reduced adhesion/ECM coupling, faster cytoskeletal dynamics, and increased possibility of force-induced adhesion retraction. By reducing the force applied at adhesion sites in a soft environment, an equilibrium is reached and results in optimal adhesion stability to promote migration. Likewise, increasing integrin activation or integrin avidity (via FN) in a soft ECM will shift the adhesion towards an equilibrium state. Thus, in a soft 3D ECM, our control condition is in a contractility-dominant state, whereas the stiff fibrils of the FB conditions promote optimal adhesion.

Where our model diverges from the two-spring model is that for fibroblasts in 3D collagen, integrin clustering does not require contractile force as is a prerequisite on a 2D surface. On a 2D ECM, we hypothesize that the integrin clustering or the binding rate is reduced to a similar degree as the unbinding rate when force at an adhesion is low. Because integrins on a native 3D fiber are able to cluster in the absence of force, the binding rate remains elevated while the unbinding rate is reduced, resulting in a “ligation dominant” scenario, which is likely the main cause of the observed migration deficits in 3D and the discrepancy with migration on 2D surfaces. This leads us to conclude that a critical balance between ECM stiffness, integrin avidity, and cytoskeletal contractility is required to optimize adhesion stability as well as cell migration in 3D (Supplementary Fig. 8b). While not studied in detail here at the adhesion level, it would be expected that shifting the adhesion population too far in the opposite “stability” direction would also

reduce cell migration, where all adhesions mature and fail to uncouple or retract from the ECM, similar to “hyper” activation of integrins when in the presence of manganese or stimulatory antibodies[3].

Supplementary Methods

Microscopy

For all live-cell fluorescence experiments, DMEM without phenol red or FluoroBrite DMEM (GIBCO) was used and supplemented with a 1:100 ratio of Oxyfluor (Oxyrase) to reduce photobleaching and phototoxicity. FBS was also reduced to 5%. Fibroblasts were imaged with a modified Yokogawa spinning-disk confocal scan head (CSU-21: modified by Spectral Applied Research, Inc.) on an automated Olympus IX-81 microscope using a 60X SAPO-Chromat silicone oil objective (N.A. 1.3) to reduce spherical aberration in 3D. A custom laser launch (built by A.D.D.) equipped with 445 nm (80 mW: Vortran Laser Technology), 488 nm (150 mW: Coherent), 514 nm (150 mW: Coherent), 568 nm (100 mW: Coherent), and 642 nm (110mW: Vortran Laser Technology) diode lasers supplied excitation wavelengths. A Gooch and Housego AOTF controlled shuttering and intensity for 488, 514, and 568 lines. 445 and 642 lines were shuttered and intensity controlled via TTL and direct voltage steps, respectively. The primary dichroics (442/568/647 and 405/488/568/647) were from Semrock (Rochester, NY). Images were captured using a backthinned EM CCD camera in 16-bit format using the 10 MHz digitization setting (Photometrics). EM gain was set between 600-800 (3X) with exposure times between70-300 ms per image taken every 30 seconds for up to 2 hours. A motorized Z-piezo stage was used to rapidly capture Z-stacks every 0.5 microns over a Z-distance of 6-20 microns (cell dependent). An environmental chamber surrounding the microscope maintained cells at a constant 37°C, with 10% $CO2$ and approximately 50% humidity (Precision Plastics, Beltsville MD). All components were controlled with MetaMorph imaging software (Molecular Devices, Downington, PA).

For FRAP experiments we used a CSU-X1 spinning disk confocal (Yokogawa, Tokyo, Japan) attached to a Zeiss Axiovert 200M microscope using a 63X Plan-Apochromat objective (NA 1.2) water immersion objective (Zeiss). An LMM5 laser merge module (Spectral Applied Research, Ontario, Canada) equipped with 405 nm (100 mW), 488 nm

(100 mW), 561(50 mW) and 642 nm (100 mW) diode lasers provided excitation wavelengths. The primary dichroic (405/488/561/640) and accompanying emission filters were from Semrock (Rochester, NY). Images were captured at 16-bits using a 512 backthinned EM CCD camera (Photometrics). X, Y, and Z positioning was performed by a MS-2000 Z-piezo stage from ASI (Eugene, OR). An environmental chamber on the microscope maintained at a constant temperature of 37°C with constant CO2 and humidity. All components were controlled with MetaMorph imaging software (Molecular Devices, Downington, PA). An iLAS FRAP module (Roper Scientific Europe, France) together with a 50 mW 405 nm diode laser (CrystaLaser, Reno, NV) attached to the epi-fluorescent port of the microscope body was used to FRAP EGFP-zyxin positive adhesions . The “FRAP on the fly” module was used with an 8 pixel diameter with an output power of 50%.

Time-lapse phase contrast images were recorded on a microscope (Axiovert 135TV; Carl Zeiss, Inc.) fitted with a motorized XY- and Z-stage focus drive (Ludl Electronic Products Ltd.) using an enhanced contrast Plan-Neofluar 10 ×0.3 NA, a long working-distance Plan-Neofluar Korr 20 × 0.4 NA, or a long working -distance Plan-Neofluar Korr 40 × 0.6 NA phase objective (Carl Zeiss, Inc.). Images were acquired with a charge-coupled device camera (ORCA II ER; Hamamatsu Photonics). MetaMorph imaging software was used to acquire images and control all hardware. A custom environmental chamber (Lucite) enclosed the microscope and maintained cells at 37°C with 10% CO_2. A red filter was used to block lower wavelengths of light during experiments using blebbistatin. SIM imaging was performed as described elsewhere[4].

Supplementary References

1. Bell, G.I. Models for the specific adhesion of cells to cells. *Science* **200**, 618-627 (1978).
2. Schwarz, U.S., Erdmann, T. & Bischofs, I.B. Focal adhesions as mechanosensors: the two-spring model. *Bio Systems* **83**, 225-232 (2006).
3. Cluzel, C. *et al.* The mechanisms and dynamics of (alpha)v(beta)3 integrin clustering in living cells. *The Journal of cell biology* **171**, 383-392 (2005).
4. York, A.G. *et al.* Resolution doubling in live, multicellular organisms via multifocal structured illumination microscopy. *Nature methods* **9**, 749-754 (2012).
